# Supplementary material for: Dissemination of 2014 dual antiplatelet therapy (DAPT) trial results: a systematic review of scholarly and media attention over 7 months
Source: BMJ Open. 2017 Nov 3;7(11):e014503. doi: 10.1136/bmjopen-2016-014503 (PMC5695450; doi:10.1136/bmjopen-2016-014503)
Supplement: Supplementary file 1 [file bmjopen-2016-014503supp001.pdf]

Appendix 1: Detail of 118 scientific communications					
**S/No**	**First Author**	**Year**	**Title**	**Journal**	**Type of scientific contribution**
1	Abo-salem	2015	Optimal duration of dual antiplatelet therapy after drug eluting stents: Meta-analysis of randomized trials	Cardiovascular Therapeutics	Article
2	Alfredsson	2015	Balancing the risks and benefits of long-term antiplatelet therapies for cardiovascular disease: clinical, research, and regulatory implications	J Am Heart Association	Editorial
3	AlJaroudi	2014	Review of Cardiovascular Literature	Journal of nuclear cardiology	Review
4	Angoulvant	2015	Dual antiplatelet therapy after acute coronary syndrome: a cardiologist-based optimal decision	Heart	Editorial
5	Aradi	2015	ATLANTIC: another reason to investigate the disconnect between stent thrombosis and mortality?	Thromosis & Haemostatis	Editorial
6	Auer	2015	Dual antiplatelet therapy duration and mortality	Lancet	Commentary
7	Becker	2015	Are at Least 12 Months of Dual Antiplatelet Therapy Needed for All Patients With Drug-Eluting Stents? Not All Patients With Drug-Eluting Stents Need at Least 12 Months of Dual Antiplatelet Therapy	Circulation	Editorial
8	Binder	2015	Duration of dual antiplatelet therapy after coronary artery stenting: where is the sweet spot between ischaemia and bleeding?	Europeaon Heart Journal	Editorial
9	Biondi-Zoccai	2015	Noncompliance and Cessation of Dual Antiplatelet Therapy After Coronary Stenting Looking at the Speck Rather Than Noticing the Log?	JACC-Cardiovascular Interventions	Editorial
10	Bonaca	2015	Long-term use of ticagrelor in patients with prior myocardial infarction	NEJM	Article
11	Brener	2015	Are at Least 12 Months of Dual Antiplatelet Therapy Needed for All Patients With Drug-Eluting Stents? All Patients With Drug-Eluting Stents Need at Least 12 Months of Dual Antiplatelet Therapy	Circulation	Editorial
12	Byrne	2015	Bioresorbable Drug-Eluting Stents: An Immature Technology in Need of Mature Application	JACC: Cardiovascular Interventions	Editorial

13	Capodanno	2015	What about the risk of thrombosis with bioresorbable scaffolds?	Eurointervention	Review
14	Capodanno	2015	Triple antithrombotic therapy in atrial fibrillation patients with acute coronary syndromes or undergoing percutaneous coronary intervention or transcatheter aortic valve replacement	Eurointervention	Editorial
15	Capodanno	2015	Impact of bridging with perioperative low-molecular-weight heparin on cardiac and bleeding outcomes of stented patients undergoing non-cardiac surgery	Thrombosis and Haemostasis	Article
16	Cassese	2015	Prolonged dual antiplatelet therapy after drug-eluting stenting: meta-analysis of randomized trials	Clinical Research in Cardiology	Article
17	Chow	2015	Drug-coated balloons: a novel advance in the percutaneous treatment of coronary and peripheral artery disease	Interventional Cardiology	Review
18	Cohen	2015	Long-term outcomes in high-risk patients with non-ST-segment elevation myocardial infarction	Journal of thrombosis and thrombolysis	Review
19	Collet	2015	Dual antiplatelet treatment after stenting–Authors' reply	The Lancet	Commentary
20	Colombo	2014	Dual Antiplatelet Therapy after Drug-Eluting Stents — How Long to Treat?	NEJM	Editorial
21	Cortese	2015	Drug-Coated Balloon angioplasty: an intriguing alternative for the treatment of Coronary Chronic Total Occlusions	International journal of cardiology	Letter
22	Costa	2015	Perspectives on the 2014 ESC/EACTS Guidelines on Myocardial Revascularization	Journal of cardiovascular translational research	Review
23	Costa	2015	Impact of clinical presentation on ischaemic and bleeding outcomes in patients receiving 6-or 24-month duration of dual-antiplatelet therapy after stent implantation: a pre-specified analysis from the PRODIGY (Prolonging Dual-Antiplatelet Treatment After Grading Stent-Induced Intimal Hyperplasia) trial	European Heart Journal	Article
24	Crea	2015	Sex differences in mechanisms, presentation and management of ischaemic heart disease	Atherosclerosis	Review
25	Cutlip	2014	Antiplatelet therapy after coronary artery stenting	UpToDate, Waltham, MA	Review

26	Curzen	2015	Prolonged antiplatelet therapy after drug-eluting stents	The Lancet	Commentary
27	de la Torre Hernandez	2015	Dual Antiplatelet Therapy for 6 Months vs 12 Months After New-generation Drug-eluting Stent Implantation: Matched Analysis of ESTROFA-DAPT and ESTROFA-2	Revista Española de Cardiología (English Edition)	Article
28	De Rango	2015	Dual Antiplatelet Therapy after Carotid Stenting: Lessons from'Big Brother'	European journal of vascular and endovascular surgery: the official journal of the European Society for Vascular Surgery	Editorial
29	Dhall	2014	Truth Vs hype	NEJM	Commentary
30	Dohan	2015	Duration of Dual Antiplatelet Therapy after Drug-Eluting Stents	NEJM	Commentary
31	Eisen	2015	Antiplatelet therapy: Defining the optimal duration of DAPT after PCI with DES	Nat Rev Cardiol	Others
32	Elmariah	2015	Extended duration dual antiplatelet therapy and mortality: a systematic review and meta-analysis	The Lancet	Article
33	Fanari	2015	Cost Effectiveness of Antiplatelet and Antithrombotic Therapy in The Setting of Acute Coronary Syndrome: current perspective and literature review	American Journal of Cardiovascular Drugs	Review
34	Fareed	2015	Antithrombotic therapy in 2014: Making headway in anticoagulant and antiplatelet therapy	Nature Reviews Cardiology	Review
35	Fiedler	2015	Duration of Triple Therapy in Patients Requiring Oral Anticoagulation After Drug-Eluting Stent Implantation The ISAR-TRIPLE Trial	Journal of the American College of Cardiology	Article
36	Genereux	2015	Stent Thrombosis and Dual Antiplatelet Therapy Interruption With Everolimus-Eluting Stents Insights From the Xience V Coronary Stent System Trials	Circulation: Cardiovascular Interventions	Article
37	Gilard	2015	Double Antiplatelet Therapy Duration: Standardize or Personalize?	Journal of the American College of Cardiology	Editorial

38	Gilchrist	2015	Vignettes of DES Failure	Catheterization and Cardiovascular Interventions	Editorial
39	Giustino	2015	Duration of Dual Antiplatelet Therapy After Drug-Eluting Stent Implantation: A Systematic Review and Meta-Analysis of Randomized Controlled Trials	Journal of the American College of Cardiology	Article
40	Gupta	2014	Balancing ischemia vs. bleeding-- Jury still out.	NEJM	Commentary
41	Gupta	2014	Dual antiplatelets :Walking on a tight rope	NEJM	Commentary
42	Habib	2015	Endothelialization of drug eluting stents and its impact on dual anti-platelet therapy duration	Pharmacol Res	Review
43	Henderson		Primecuts–This Week In The Journals	Clinical Correlations	Others
44	Hernandez	2015	2014 Update on Interventional Cardiology	Revista Española de Cardiología	Review
45	Huang	2015	Is the Duration of Dual Antiplatelet Therapy after Implantation of Drug-Eluting Stents the Longer the Better	Medical Principles and Practice	Letter
46	Husted	2015	Antithrombotic therapy for long-term secondary prevention of acute coronary syndrome in high-risk patients	Therapeutics and clinical risk management	Review
47	Huynh	2015	Antiplatelet therapy: Risks and benefits of extended DAPT after stenting	Nat Rev Cardiol	Others
48	Iqbal	2015	The year in cardiology 2014: coronary intervention	European Heart Journal	Review
49	Kumana	2015	Absolute benefits and harms of dual antiplatelet therapy after drug eluting stenting	Hong Kong Medical Journal	Article
50	Keaney	2015	Balancing the Risks and Benefits of Dual Platelet Inhibition	NEJM	Editorial

51	Kereiakes	2015	Efficacy and Safety of a Novel Bioabsorbable Polymer-Coated, Everolimus-Eluting Coronary Stent The EVOLVE II Randomized Trial	Circulation-Cardiovascular Interventions	Article
52	Kereiakes	2015	Antiplatelet therapy duration following bare metal or drug-eluting coronary stents: The dual antiplatelet therapy randomized clinical trial	JAMA	Article
53	Kirtane	2015	Should all stent patients have prolonged dual antiplatelet therapy?	JACC: Cardiovascular Interventions	Editorial
54	Kohno	2015	Report of the American Heart Association (AHA) Scientific Sessions 2014, Chicago	Circulation Journal	Commentary
55	Koppara	2015	Optical coherence tomography surveillance following drug-eluting stent implantation	Minerva Cardioangiologica	Review
56	Lavi	2015	Biodegredable stent platforms–Are we heading in the right direction?	Canadian Journal of Cardiology	Editorial
57	Lee	2015	Bleeding risks are in the eye of the beholder	ACP Journal Club	Commentary
58	Lee	2014	Dual Antiplatelet Therapy for Coronary Artery Disease	Circulation Journal	Review
59	Lemesle	2015	Dual antiplatelet therapy and non-cardiovascular mortality	The Lancet	Commentary
60	Lhermusier	2015	Prasugrel hydrochloride for the treatment of acute coronary syndromes	Expert opinion on pharmacotherapy	Review
61	Liou	2015	Optimal duration of dual antiplatelet therapy following drug-eluting stents implantation: A meta-analysis of 7 randomised controlled trials	International journal of cardiology	Article
62	Lipkin	2014	1 out of a hundred patient will benefit from extended dual Rx	NEJM	Commentary
63	Liu	2015	P2Y12 receptor inhibitors for secondary prevention of ischemic stroke	Expert opinion on pharmacotherapy	Review
64	Liu	2015	Percutaneous coronary intervention strategies and prognosis for graft lesions	Experimental and	Article

			following coronary artery bypass grafting	Therapeutic Medicine	
65	Madhavan	2015	Post-PCI Antithrombotic Therapy in Patients Requiring Long-Term Anticoagulation	Current cardiology reports	Review
66	Marrs	2015	Duration of Dual Antiplatelet Therapy after Drug-Eluting Stents	NEJM	Commentary
67	Matteau	2015	Balancing Long-Term Risks of Ischemic and Bleeding Complications after Percutaneous Coronary Intervention with Drug-Eluting Stents	The American journal of cardiology	Article
68	Matthews	2015	Persistence with secondary prevention medications after acute myocardial infarction: Insights from the TRANSLATE-ACS study	American Heart Journal	Article
69	Mauri	2015	Duration of Dual Antiplatelet Therapy after Drug-Eluting Stents -Author's reply	NEJM	Commentary
70	McKavanagh	2015	A Review of the Key Clinical Trials of 2014	Cardiology and therapy	Review
71	McMillan	2014	Nice slant	New England Journal of Medicine	Commentary
72	Mega	2015	Pharmacology of antithrombotic drugs: an assessment of oral antiplatelet and anticoagulant treatments	The Lancet	Review
73	Mehran	2015	DAPT Duration After DES: What Is the “Mandatory” Duration?	Journal of the American College of Cardiology	Editorial
74	Meneses	2014	About DAPT trial	New England Journal of Medicine	Commentary
75	Moschonas	2015	Protease-activated receptor-1 antagonists in long-term antiplatelet therapy. Current state of evidence and future perspectives	International journal of cardiology	Review

76	Mukherjee	2015	After drug-eluting stent placement, 6 months of dual antiplatelet therapy was noninferior to 12 months	Annals of Internal Medicine	Commentary
77	Navarese	2015	Optimal duration of dual antiplatelet therapy after percutaneous coronary intervention with drug eluting stents: meta-analysis of randomised controlled trials	BMJ	Article
78	Palmerini	2015	Mortality in patients treated with extended duration dual antiplatelet therapy after drug-eluting stent implantation: a pairwise and Bayesian network meta-analysis of randomised trials	The Lancet	Article
79	Papadimitriou	2015	Triple Antithrombotic Therapy: Is it Time to Drop the Aspirin?	Hospital Chronicles	Review
80	Parmar	2014	Error in Study Procedures!	NEJM	Commentary
81	Price	2015	The Optimal Duration of Dual Antiplatelet Therapy After Drug-Eluting Stent Implantation: Chasing a Mirage	Journal of the American College of Cardiology	Editorial
82	Raffoul	2015	Dual antiplatelet therapy duration after the placement of a drug-eluting stent: what are the data?	Current treatment options in cardiovascular medicine	Review
83	Rao	2015	The Conundrum of Reducing Ischemic and Bleeding Events After PCI∗	Journal of the American College of Cardiology	Editorial
84	Reejhsinghani	2015	Prevention of stent thrombosis: challenges and solutions	Vasc Health Risk Manag	Review
85	Rinfret	2015	Percutaneous Coronary Intervention: Finally Mature Enough	Journal of the American College of Cardiology	Editorial
86	Robbins	2015	Periprocedural management of aspirin during colonoscopy: a survey of practice patterns in the United States	Gastrointestinal endoscopy	Article

87	Rohla	2015	Double or triple antithrombotic combination therapy in patients who need anticoagulation and antiplatelet therapy in parallel	European Heart Journal-Cardiovascular Pharmacotherapy	Review
88	Ruparelia	2015	Dual antiplatelet therapy following drug-eluting stent implantation: how long is long enough?	Expert review of cardiovascular therapy	Editorial
89	Sabouret	2015	Dual antiplatelet therapy: optimal timing, management, and duration	European Heart Journal-Cardiovascular Pharmacotherapy	Review
90	Samardzic	2015	Temporal changes of platelet reactivity after coronary stenting–a thing to think about	American Journal of Cardiology	Commentary
91	Schiele	2015	mpact of prolonged dual antiplatelet therapy after acute myocardial infarction on 5-year mortality in the FAST-MI 2005 registry	International journal of cardiology	Article
92	Schulz-Schupke	2015	ISAR-SAFE: a randomized, double-blind, placebo-controlled trial of 6 vs. 12 months of clopidogrel therapy after drug-eluting stenting	European Heart Journal	Article
93	Secemsky	2015	Comparison of Short-and Long-Term Cardiac Mortality in Early Versus Late Stent Thrombosis (from Pooled PROTECT Trials)	The American journal of cardiology	Article
94	Shimohama	2015	Intrastent Thrombus - What You See Is What You Get?	Circulation Journal	Editorial
95	Simon	2015	Omeprazole, pantoprazole, and CYP2C19 effects on clopidogrel pharmacokinetic-pharmacodynamic relationships in stable coronary artery disease patients	European Journal of Clinical Pharmacology	Article
96	Sipahi	2015	Duration of Dual Antiplatelet Therapy after Drug-Eluting Stents	NEJM	Commentary
97	Sommer	2015	Stent Thrombosis: Current Management and Outcomes	Current treatment options in cardiovascular medicine	Review

98	Spencer	2015	Dual antiplatelets for 30 mo after drug-eluting stents reduced stent thrombosis and CV and cerebrovascular events.	ACP Journal Club	Commentary
99	Spencer	2015	Longer Versus Shorter Duration Dual-Antiplatelet Therapy After Drug-Eluting Stent PlacementA Systematic Review and Meta-analysisDuration of Dual-Antiplatelet Therapy After Drug-Eluting Stents	Annals of Internal Medicine	Article
100	Takeuchi	2015	Optimum duration of dual antiplatelet treatment could be decided using 64-MDCT: A new hint to treating patients with stents	IJC Heart & Vasculature	Others
101	Thomas	2015	The future of P2Y12 receptor antagonists	Platelets	Review
102	Tomoda	2015	Duration of Dual Antiplatelet Therapy after Drug-Eluting Stents	NEJM	Commentary
103	Toyota	2015	Meta-analysis of Long-term Clinical Outcomes of Everolimus-eluting Stents	The American journal of cardiology	Article
104	Tremmel	2015	Late breaking trials of 2014 in coronary artery disease: Commentary covering ACC, EuroPCR, SCAI, TCT, ESC, and AHA	Catheterization and Cardiovascular Interventions	Commentary
105	Tsoumani	2015	Evaluating the bioequivalence of clopidogrel generic formulations	Current medical research and opinion	Editorial
106	Valgimigli	2015	Duration of dual antiplatelet therapy after drug-eluting stent implantation: will we ever reach a consensus?	European Heart Journal	Editorial
107	Van de Werf	2015	The year in cardiology 2014: acute coronary syndromes	European Heart Journal	Review
108	Vetrovec	2015	Another Challenge for the Presumed Safety Advantage of Bare Metal Stents	Catheterization and Cardiovascular Interventions	Editorial
109	Vranckx	2015	Peri-procedural use of rivaroxaban in elective percutaneous coronary intervention to treat stable coronary artery disease. The XPLORER trial	Thrombosis and Haemostasis	Article
110	Waksman	2015	Do you still have an appetite for a short DAPT trial?	Cardiovascular Revascularization Medicine	Editorial

111	Watanabe	2015	Antiplatelet therapy discontinuation and the risk of serious cardiovascular events after coronary stenting: observations from the CREDO-Kyoto Registry Cohort-2	PLoS ONE	Article
112	Wiviott	2015	Clinical evidence for oral antiplatelet therapy in acute coronary syndromes	The Lancet	Review
113	Yamaji	2015	Long-term Outcomes after Coronary Stent Implantation in Patients Presenting with versus without Acute Myocardial Infarction (An observation from Coronary Revascularization Demonstrating Outcome Study-Kyoto Registry Cohort-2)	The American journal of cardiology	Article
114	Yang	2015	Current antiplatelet agents: place in therapy and role of genetic testing	Journal of thrombosis and thrombolysis	Review
115	Yeh	2015	Benefits and risks of extended duration dual antiplatelet therapy after PCI in patients with and without acute myocardial infarction	Journal of the American College of Cardiology	Article
116	Yeh	2015	Dual Antiplatelet Platelet Therapy Duration Following Coronary Stenting	Journal of the American College of Cardiology	Editorial
117	Yeh	2015	Dual antiplatelet therapy duration and mortality–Authors' reply	The Lancet	Commentary
118	Yeh	2015	Close encounters with errors of the second kind: evaluating risks and benefits of long-term dual antiplatelet therapy	European Heart Journal	Editorial
