# Supplementary material for: Dissemination of 2014 dual antiplatelet therapy (DAPT) trial results: a systematic review of scholarly and media attention over 7 months
Source: BMJ Open. 2017 Nov 3;7(11):e014503. doi: 10.1136/bmjopen-2016-014503 (PMC5695450; doi:10.1136/bmjopen-2016-014503)
Supplement: Supplementary file 2 [file bmjopen-2016-014503supp002.pdf]

Appendix 2: Content of the scholarly and public attention surrounding the DAPT study by source (n = 425)

Category	Overall n=425	Scientific communication 118 (27.7)	News 12 (2.8)	Blogs 3 (0.7)	Facebook posts 189 (44.4)	Tweets 75 (17.6)	YouTube 8 (1.9)	DAPT Website 20 (4.7)
Text favourable about the prolonged treatment	81 (19.1)	28 (23.7)	9 (75.0)	-	9 (4.8)	11 (14.7)	(100)	16 (80.0)
Text uncertain, with inappropriate mention of mortality	19 (4.5)	13 (11.0)	3 (25.0)	2 (66.7)	-	-	-	1 (5.0)
Electronic link	151 (35.5)	-	-	-	113 (59.8)	38 (50.6)	-	-
Referenced with no message	13 (3.1)	1 (0.8)	-	-	10 (5.3)	2 (2.7)	-	-
Text uncertain, with no mention of mortality	100 (23.5)	37 (31.4)	-	1 (33.3)	48 (25.4)	13 (17.3)	-	1 (5.0)
Text uncertain, with appropriate mention of mortality	34 (8.0)	29 (24.6)	-	-	1 (0.5)	3 (4.0)	-	1 (5.0)
Text not favourable about the prolonged treatment	15 (3.5)	3 (2.5)	-	-	8 (4.2)	3 (4.0)	-	1 (5.0)
Text not favourable about the prolonged treatment and critical of the authors' interpretation	12 (3.0)	7 (6.0)	-	-	-	5 (6.7)	-	-
