# Supplementary material for: Dissemination of 2014 dual antiplatelet therapy (DAPT) trial results: a systematic review of scholarly and media attention over 7 months
Source: BMJ Open. 2017 Nov 3;7(11):e014503. doi: 10.1136/bmjopen-2016-014503 (PMC5695450; doi:10.1136/bmjopen-2016-014503)
Supplement: Author's manuscript [file bmjopen-2016-014503.draft_revisions.pdf]

BMJ Open is committed to open peer review. As part of this commitment we make the peer review history of every article we publish publicly available.

When an article is published we post the peer reviewers' comments and the authors' responses online. We also post the versions of the paper that were used during peer review. These are the versions that the peer review comments apply to.

The versions of the paper that follow are the versions that were submitted during the peer review process. They are not the versions of record or the final published versions. They should not be cited or distributed as the published version of this manuscript.

BMJ Open is an open access journal and the full, final, typeset and author-corrected version of record of the manuscript is available on our site with no access controls, subscription charges or pay-per-view fees (http://bmjopen.bmj.com).

If you have any questions on BMJ Open's open peer review process please email editorial.bmjopen@bmj.com

BMJ Open

Lack of critical opinion to highlight misleading interpretation of increased risk of mortality in the Dual Anti-platelet Therapy (DAPT) study

Journal:	*BMJ Open*
Manuscript ID	bmjopen-2016-014503
Article Type:	Research
Date Submitted by the Author:	28-Sep-2016
Complete List of Authors:	Sharp, Melissa K; Columbia University, Mailman School of Public Health; INSERM UMR 1153, Center of Research in Epidemiology and Statistics Sorbonne Paris Cité, Center of Epidemiology and Clinical Research<br>HANEEF, Romana; INSERM UMR 1153, Center of Research in Epidemiology and Statistics Sorbonne Paris Cité, Center of Epidemiology and Clinical Research; University of Paris Descartes<br>Ravaud, Philippe; INSERM UMR 1153, Center of Research in Epidemiology and Statistics Sorbonne Paris Cité, Center of Epidemiology and Clinical Research; University of Paris Descartes<br>Boutron, Isabelle; INSERM UMR 1153, Center of Research in Epidemiology and Statistics Sorbonne Paris Cité, Center of Epidemiology and Clinical Research; University of Paris Descartes
**Primary Subject Heading**:	Epidemiology
Secondary Subject Heading:	Qualitative research, Public health, Research methods, Communication
Keywords:	DAPT Therapy, Misleading interpretation, Mortality, Public attention, Critical opinion

SCHOLARONE™
Manuscripts

Lack of critical opinion to highlight misleading interpretation of increased risk of mortality in the Dual Anti-platelet Therapy (DAPT) study

Melissa K Sharp• (1,2), Romana Haneef• (2,3,4), Philippe Ravaud (1,2,3,4,5), Isabelle Boutron*(2,3,4,5)

(1) Columbia University, Mailman School of Public Health, New York, NY, USA

(2) METHODS Team, Center of Research in Epidemiology and Statistics Sorbonne Paris Cité, UMR 1153, INSERM, Paris, France.

(3) University Paris Descartes, Sorbonne Paris Cité, Paris, France

(4) Centre d'Épidémiologie Clinique, AP-HP (Assistance Publique des Hôpitaux de Paris), Hôpital Hôtel Dieu, Paris, France

(5) Cochrane France, Paris, France

• These authors contributed equally to this work as a first author.

*Corresponding author:

Isabelle Boutron

Centre d'Epidémiologie Clinique

Hôpital Hôtel Dieu,

1, Place du parvis Notre Dame

75004 Paris Cedex 4

E-mail : isabelle.boutron@aphp.fr

Tel : 00 33 1 42 34 78 33

Fax : 00 33 1 42 34 87 90

For peer review only - http://bmjopen.bmj.com/site/about/guidelines.xhtml

Abstract

Objective: To explore how the results from DAPT Trial 2014, were disseminated to the scientific community and public.

Design: A cross-sectional study of scholarly and public attention surrounding DAPT study.

Settings: Data were collected from following sources: ISI Web of Knowledge, Google Scholar, PubMed Commons, EurekAlert, the DAPT study website (www.daptstudy.org), and the *New England Journal of Medicine* website *(for scholarly attention)* and Altmetric Explorer, Snap Bird, YouTube *(for public attention)* citing DAPT study results appearing from November 16, 2014 to June 10, 2015.

Participants: No participants were involved in this study.

Main outcome measure: Proportion of contents highlighting the increased risk of mortality and critical to the author's questionable interpretation of the results.

Results: We identified 425 items reported by 7 sources; 164 (39%) disseminated the authors' questionable interpretation via an electronic link or a reference, with no additional text. Among 81 items (19%), the message favoured prolonged treatment and consequently overstated the article conclusions. Among 119 items (28%), the text was uncertain about the benefit of prolonged treatment but was reported with no or inappropriate mention of increased risk of mortality. Only 34 items (8%) were uncertain about the benefit of prolonged treatment and appropriately mentioned increased risk of mortality. 27 (6%) did not favour prolonged treatment, of which only 12 (3%) clearly raised some concerns about the reporting of increased risk of death.

Conclusion: The amount of contents criticizing DAPT study authors' questionable interpretation, particularly related to increased risk of mortality was limited.

For peer review only - http://bmjopen.bmj.com/site/about/guidelines.xhtml

Strengths and limitation of this study

- Our method involved a broad search strategy, which ensured to capture an extensive and representative sample of contents citing DAPT trial for both scholarly and public attention.
- Our systematic approach to analyze the text of contents provides a comprehensive overview of dissemination of this study results.
- This study only focussed on a specific trial publication and results are not generalizable to other studies.

For peer review only

For peer review only - http://bmjopen.bmj.com/site/about/guidelines.xhtml

INTRODUCTION

Development of optimal coronary stent replacement has progressed rapidly over recent years [1]. In the United States, almost 700,000 stents are placed every year and there is an increasing trend in Europe in its use [2]. Dual antiplatelet therapy (DAPT) (i.e., P2Y12-receptor inhibitor combined with aspirin) is recommended after placement of coronary stents to prevent thrombotic complications [3]. The optimal duration of DAPT has been debated [4-8].

In December, 2014, the Harvard Clinical Research Institute (HCRI) released the results of the DAPT study, the largest international randomized controlled trial to date [9]. The trial aimed to determine the benefits and risks of continuing DAPT beyond 1 year after placement of a coronary stent [9]. A total of 9,961 adult patients were randomly assigned to continue thienopyridine treatment or to receive a placebo for 30 months. Continued therapy reduced the rates of stent thrombosis (0.4% vs.1.4%; $p<0.001$) and major adverse cardiovascular and cerebrovascular events (MACCEs) (2.1% vs. 4.1%; $p<0.001$) with an expected increase in the rate of moderate or severe bleeding (2.5% vs. 1.6%; $p=0.001$) [9]. However, continued therapy was also associated with an increase of 36% in all-cause mortality (2.0% vs. 1.5%; hazard ratio 1.36 [95% CI, 1.00 to 1.85]; $P=0.05$).

The results of the DAPT study were published in the *New England Journal of Medicine* (*NEJM*) [9] after the presentation of results at the American Health Association Conference, in November 2014. However, the reporting of the results raised some concerns [10, 11]. Particularly, the abstract conclusions did not mention the increased risk of mortality. Further, the discussion included questionable explanations based on post-hoc analyses to clear the role of prolonged thienopyridine treatment on this increased risk of mortality. For this purpose, the authors had split the analysis by cause of death, which reduced the power to show a statistically significant difference. Then, they focused on the increase in cancer-related death

For peer review only - http://bmjopen.bmj.com/site/about/guidelines.xhtml

(0.62% vs 0.28%, p=0.02). However, instead of raising the hypothesis that prolonged treatment could increase the risk of cancer or the risk of dying from cancer, they interpreted this finding as being related to an imbalance at baseline in patients with a history of cancer before enrollment (9.8% vs 9.5%). To confirm this hypothesis, the authors performed a post-hoc analysis excluding all deaths that could be related to cancer diagnosed before enrolment. This post-hoc exclusion of patients with an event is a concern. As expected, the results became statistically non-significant (0.50% vs 0.28%, p=0.11). The authors did not mention other studies showing that prasugrel, one thienopyridine used in this trial, has been associated with a significantly increased risk of incident cancer [12] and has been specifically investigated by the US Food and Drug Administration [13].

Here we aimed to explore how these results from the DAPT trial were disseminated to the scientific community and the public. Particularly, we aimed to determine whether the scholarly and public attention raised by this study highlighted the increased risk of mortality and criticized the authors' questionable interpretation of the findings.

For peer review only - http://bmjopen.bmj.com/site/about/guidelines.xhtml

METHODS

We performed a cross-sectional study of scholarly and public attention surrounding DAPT study.

Identification of scholarly and public attention surrounding DAPT study

Scholarly attention

On June 2015, we searched the following electronic databases to identify responses to the DAPT study: ISI Web of Knowledge, Google Scholar, and PubMed Commons, and Comment. We also searched the comments and citing articles on the *NEJM* webpage for the original article [9].

Public attention

We searched Altmetric Explorer [14-17] to identify all online attention (news, blogs, Twitter, Facebook, Google+, Mendeley, CiteULike) given to the DAPT study. Each identified social media source was then systematically evaluated to determine whether there were other posts that were not captured by Altmetric Explorer. In addition, each original tweet was reviewed to find retweets, replies and favorites. Since Altmetric.com captures only tweets attached to the DOI (Digital Object Identifier) of the original DAPT article, we also used snapbird.org, a search engine that can search an individual twitter account by using the *NEJM*'s Twitter account and the search terms "DAPT" and "dual antiplatelet therapy". We also searched EurekAlert! (an online free database for science press releases, www.eurekalert.org) for press releases dedicated to the DAPT study, YouTube (search terms "DAPT" and "dual antiplatelet therapy"); and pages dedicated to patients, clinicians and media at the DAPT study website (http://www.daptstudy.org).

For peer review only - http://bmjopen.bmj.com/site/about/guidelines.xhtml

Eligibility criteria

Two researchers (MS & RH) screened all items retrieved and selected all English-language items that cited the DAPT study and were released from November 16, 2014 to June 10, 2015. Any disagreement was resolved by consensus.

Content of scholarly and public attention surrounding DAPT study

Two researchers (MS, RH) read the items from each source independently and evaluated them by using a preliminarily tested extraction form. Disagreements were resolved by discussion until consensus was reached. If needed, a third researcher (IB) appraised the content.

We determined whether the source consisted of a reference or a link to the *NEJM* article reporting the DAPT study only or was a text commenting on the DAPT study. For a text commenting on the DAPT study, we checked whether the original study authors were involved in writing the text or not. Our main outcome of interest was the proportion of contents highlighting the increased risk of mortality and critical to the author's questionable interpretation of the results. We determined whether;

- the primary efficacy outcomes (i.e., stent thrombosis and MACCE) were reported
- the safety outcomes related to moderate or severe bleeding were reported
- the increased risk of mortality with prolonged treatment was reported
- the authors' questionable explanation clearing the responsibility of prolonged treatment in the increased risk of mortality was reported or criticized.
- the content of the text was 1) favourable about the prolonged treatment and consequently overstating the article conclusion, 2) uncertain about the benefit of the prolonged treatment (i.e., statement of both the beneficial effect, and increased risk of bleeding, text ending with a question mark, use of "may or might" or reporting that the study needs further research), or 3) not favourable about the prolonged treatment [18].

For peer review only - http://bmjopen.bmj.com/site/about/guidelines.xhtml

Overall, we classified the sources based on the text of contents as follows:

1. Text favourable towards the prolonged treatment
2. Text uncertain (about the benefit of prolonged treatment) with inappropriate mention of mortality
3. Text neutral/uncertain (about the benefit of prolonged treatment) with no mention of mortality
4. Electronic link or referenced with no message
5. Text uncertain (about the benefit of prolonged treatment) with appropriate mention of mortality
6. Text not favourable about the prolonged treatment
7. Text not favourable about the prolonged treatment and critical of the authors' interpretation

Statistical analysis

We calculated frequencies and percentages (%) for qualitative variables and median (interquartile range) for quantitative variables.

For peer review only

For peer review only - http://bmjopen.bmj.com/site/about/guidelines.xhtml

RESULTS

Identification of scholarly and public attention surrounding DAPT study

From all sources, we selected and appraised 425 items: 118 communications, 12 news items, 3 blogs, 189 Facebook posts or comments, 75 tweets or replies, 8 videos on YouTube, 14 DAPT media pages, 5 DAPT website pages and 1 video on the DAPT website (Figure 1). The original study authors were directly involved in 35 items.

Reporting of the content

The items are described in Figures 2 and 3. Overall, 164 items (39%) involved disseminating the authors' questionable reporting and interpretation via an electronic link (n=151, 36%) or reference (n=13; 3%), with no additional text or message. Among 81 items (19%), the message favoured the prolonged treatment and therefore overstated the article conclusions. For example, the DAPT study website dedicated to patients reported that "*It is important that patients who currently take a thienopyridine anti-clotting medication (clopidogrel or prasugrel) do not stop taking their medication. […] The benefits of continuing dual antiplatelet therapy for one year, according to current guidelines, far outweigh the risks*." Among 119 items (28%), the text was uncertain about the benefit of prolonged treatment but was reported with no mention of the increased risk of mortality (100, 24%) or the questionable explanation clearing the responsibility of prolonged treatment (n=19; 4%). Overall, 34 items (8%) were uncertain about the benefit of prolonged treatment but mentioned the increased risk of mortality. Only 27 (6%) did not favour prolonged treatment and only 12 of these (3%) clearly raised some concerns about the reporting of the increased risk of death. Further information on items by source is in *appendix 1.*

Overall, 136 (32%) items reported efficacy outcomes (i.e., stent thrombosis and MACCEs), 127 (30%) safety outcomes and 113 (27%) both efficacy and safety outcomes.

For peer review only - http://bmjopen.bmj.com/site/about/guidelines.xhtml

Overall, 100 items (24%) did not mention mortality, but when mortality was mentioned, in 19 items (5%), it was reported with the authors' questionable justification for prolonged treatment.

For peer review only

For peer review only - http://bmjopen.bmj.com/site/about/guidelines.xhtml

DISCUSSION

We described the dissemination of the DAPT study findings in scientific journals and to the public via different sources such as news, blogs, and social media. Our assessment of 425 items disseminating the DAPT study results showed that only 8% of the items mentioned some uncertainty about the benefit of prolonged treatment and included an appropriate mention of the increased risk of mortality. Furthermore, only 12 items (3%) clearly raised some concerns about the reporting of the increased risk of death. This study adds to the burgeoning literature on the biased dissemination of research results. Previous studies have focused on publication bias [19], selective reporting of outcomes [19-24], and spin [21, 25, 26].

However, this is the first study to our knowledge to focus on both scholarly and public dissemination of study results. Our approach involved a broad search strategy and multiple search engines, which ensured to capture an extensive and representative sample of contents discussing DAPT study results. Each social media item from Altmetric was systematically reviewed for additional content that may have been missed, and several different search engines were used. We captured items that were published over the course of many months, which highlighted the perpetuation and continuation of the dissemination of the questionable interpretations. The inclusion period for sources seemed to be more than sufficient because tweets linked to scientific articles have been shown to taper off well before our cutoff point (7 months) [27]. Additionally, two independent researchers assessed each source by using a standardized data extraction form and disagreements were resolved by consensus.

However, our study is not without limitations. This study only focussed on a specific trial publication and results are not generalizable to other studies. Although the article we focused on was among the top 5 of all research outputs and the 99th percentile of articles on Altmetric. The data extraction involved some subjectivity; however, we tried to address this by using a standardized data extraction form and independent assessment as well as consensus among

For peer review only - http://bmjopen.bmj.com/site/about/guidelines.xhtml

two researchers. Finally, despite our best efforts, we cannot ensure that our search strategy was all-encompassing because of the breadth of social media.

This analysis raises important concerns related to the impact of prolonged treatment with DAPT on the risk of death. After the publication of this trial [9], several meta-analyses with contradictory results were published in 2015. First, researchers involved in DAPT trial concluded in a meta-analysis published in *The Lancet* that prolonged DAPT duration was not associated with a difference in risk of all-cause mortality [28]. However, in this meta-analysis, the authors did not use a consistent definition of prolonged DAPT treatment. The authors pooled results from a study that defined 12 months of treatment as short DAPT and one that defined 12 months of treatment as prolonged DAPT. The 3 meta-analyses, published by different teams, showed that prolonged DAPT was associated with increased risk of all-cause mortality [4, 5, 8].

CONCLUSIONS

Dissemination of the DAPT study results for scientists and the public in different media sources rarely criticized the authors' questionable conclusions and interpretation of the results, particularly related to increased risk of mortality.

Supplementary Data

Appendix 1: Content of scholarly and public attention surrounding DAPT study by source.

For peer review only

Acknowledgements

We thank Elise DIARD for help in creating Figure 2. We acknowledge support from Altmetric for free access to "Altmetric Explorer". Isabelle Boutron and Philippe Ravaud submitted a letter to the *NEJM* following the publication of the DAPT study to highlight the inadequate reporting in the abstract conclusions, but the letter was rejected.

Contributors

Conceived, designed, selection of contents and data extraction: MS, RH. Study conception and design: MS, RH, IB. Selection of contents, data extraction: MS, RH. Analysis of data and interpretation of results: RH, PR, IB. Contributed to the writing of the manuscript: MS, RH, PR, IB. All authors read and approved the final manuscript.

Funding Source

This study did not receive any funding.

Competing interests

None declared.

Ethical approval

Not needed

Data sharing

All relevant data are included in this manuscript. Details of text content are available upon request for academic researchers.

For peer review only

Open Access

This is an Open Access article distributed in accordance with the Creative Commons Attribution Non Commercial (CC BY-NC 4.0) license, which permits others to distribute, remix, adapt, build upon this work non-commercially, and license their derivative works on different terms, provided the original work is properly cited and the use is non-commercial. See: http://creativecommons.org/licenses/by-nc/4.0/.

For peer review only

For peer review only - http://bmjopen.bmj.com/site/about/guidelines.xhtml

References

1. Wilson WM. Advances in coronary stent technology: current expectations and new developments. *Research Reports in Clinical Cardiology* 2013.
2. Mozaffarian D, Benjamin EJ, Go AS, et al. Heart disease and stroke statistics--2015 update: a report from the American Heart Association. *Circulation* 2015; **131**: e29-322.
3. Palmerini T, Dangas G, Mehran R, et al. Predictors and Implications of Stent Thrombosis in Non–ST-Segment Elevation Acute Coronary Syndromes: The ACUITY Trial. *Circulation: Cardiovascular Interventions* 2011; **4**: 577-84.
4. Palmerini T, Benedetto U, Bacchi-Reggiani L, et al. Mortality in patients treated with extended duration dual antiplatelet therapy after drug-eluting stent implantation: a pairwise and Bayesian network meta-analysis of randomised trials. *The Lancet* 2015.
5. Spencer FA, Prasad M, Vandvik PO, Chetan D, Zhou Q, Guyatt G. Longer- Versus Shorter-Duration Dual-Antiplatelet Therapy After Drug-Eluting Stent PlacementA Systematic Review and Meta-analysisDuration of Dual-Antiplatelet Therapy After Drug-Eluting Stents. *Annals of Internal Medicine* 2015; **163**: 118-26.
6. Levine GN, Bates ER, Blankenship JC, et al. 2011 ACCF/AHA/SCAI Guideline for Percutaneous Coronary InterventionA Report of the American College of Cardiology Foundation/American Heart Association Task Force on Practice Guidelines and the Society for Cardiovascular Angiography and Interventions. *Journal of the American College of Cardiology* 2011; **58**: e44-e122.
7. Windecker S, Kolh P, Alfonso F, et al. 2014 ESC/EACTS Guidelines on myocardial revascularization. *The Task Force on Myocardial Revascularization of the European Society of Cardiology (ESC) and the European Association for Cardio-Thoracic Surgery (EACTS)Developed with the special contribution of the European Association of Percutaneous Cardiovascular …* 2014; **35**: 2541-619.
8. Navarese EP, Andreotti F, Schulze V, et al. Optimal duration of dual antiplatelet therapy after percutaneous coronary intervention with drug eluting stents: meta-analysis of randomised controlled trials. *British Medical Journal* 2015; **350**.
9. Mauri L, Kereiakes DJ, Yeh RW, et al. Twelve or 30 Months of Dual Antiplatelet Therapy after Drug-Eluting Stents. *New England Journal of Medicine* 2014; **371**: 2155-66.
10. Lipkin D. 1 out of a hundred patient will benefit from extended dual Rx. 2014.
11. Gupta R. Dual antiplatelets :Walking on a tight rope. 2014.
12. Wiviott SD, Braunwald E, McCabe CH, et al. Prasugrel versus Clopidogrel in Patients with Acute Coronary Syndromes. *New England Journal of Medicine* 2007; **357**: 2001-15.
13. Unger EF. Weighing Benefits and Risks — The FDA's Review of Prasugrel. *New England Journal of Medicine* 2009; **361**: 942-5.
14. Altmetric, https://www.altmetric.com/whatwedo.php. 2012.
15. Haustein S, Costas R, Larivière V. Characterizing Social Media Metrics of Scholarly Papers: The Effect of Document Properties and Collaboration Patterns. *PLoS ONE* 2015; **10**: e0120495.
16. Liu CL, Xu YQ, Wu H, Chen SS, Guo JJ. Correlation and Interaction Visualization of Altmetric Indicators Extracted From Scholarly Social Network Activities: Dimensions and Structure. *Journal of Medical Internet Research* 2013; **15**: e259.

For peer review only - http://bmjopen.bmj.com/site/about/guidelines.xhtml

17. Thelwall M, Haustein S, Larivière V, Sugimoto CR. Do Altmetrics Work? Twitter and Ten Other Social Web Services. *PLoS ONE* 2013; **8**: e64841.
18. Yank V, Rennie D, Bero LA. Financial ties and concordance between results and conclusions in meta-analyses: retrospective cohort study. *BMJ* 2007; **335**: 1202-5.
19. Dwan K, Altman DG, Arnaiz JA, et al. Systematic Review of the Empirical Evidence of Study Publication Bias and Outcome Reporting Bias. *PLoS ONE* 2008; **3**: e3081.
20. Riveros C, Dechartres A, Perrodeau E, Haneef R, Boutron I, Ravaud P. Timing and Completeness of Trial Results Posted at ClinicalTrials.gov and Published in Journals. *PLoS Med* 2013; **10**: e1001566.
21. Boutron I, Dutton S, Ravaud P, Altman DG. Reporting and interpretation of randomized controlled trials with statistically nonsignificant results for primary outcomes. *JAMA* 2010; **303**.
22. Tang E, Ravaud P, Riveros C, Perrodeau E, Dechartres A. Comparison of serious adverse events posted at ClinicalTrials.gov and published in corresponding journal articles. *BMC Medicine* 2015; **13**: 1-8.
23. Mathieu S, Boutron I, Moher D, Altman DG, Ravaud P. Comparison of registered and published primary outcomes in randomized controlled trials. *JAMA* 2009; **302**.
24. Chan A, Hróbjartsson A, Haahr MT, Gøtzsche PC, Altman DG. Empirical evidence for selective reporting of outcomes in randomized trials: Comparison of protocols to published articles. *JAMA* 2004; **291**: 2457-65.
25. Lazarus C, Haneef R, Ravaud P, Boutron I. Classification and prevalence of spin in abstracts of non-randomized studies evaluating an intervention. *BMC Medical Research Methodology* 2015; **15**: 1-8.
26. Yavchitz A, Boutron I, Bafeta A, et al. Misrepresentation of Randomized Controlled Trials in Press Releases and News Coverage: A Cohort Study. *PLoS Med* 2012; **9**: e1001308.
27. Eysenbach G. Can Tweets Predict Citations? Metrics of Social Impact Based on Twitter and Correlation with Traditional Metrics of Scientific Impact. *J Med Internet Res* 2011; **13**: e123.
28. Elmariah S, Mauri L, Doros G, et al. Extended duration dual antiplatelet therapy and mortality: a systematic review and meta-analysis. *The Lancet* 2015; **385**: 792-8.

For peer review only - http://bmjopen.bmj.com/site/about/guidelines.xhtml

Figure 1: Flow diagram of identified scholarly and public attention surrounding DAPT study

For peer review only

For peer review only - http://bmjopen.bmj.com/site/about/guidelines.xhtml

Figure 2: Content of scholarly and public attention surrounding DAPT study (n = 425)

For peer review only

*Increased risk of mortality reported with the authors' questionable explanation clearing the responsibility of prolonged treatment in the increased risk

** Increased risk of mortality reported without any explanation

For peer review only - http://bmjopen.bmj.com/site/about/guidelines.xhtml

Figure 3: Content of scholarly and public attention surrounding DAPT study by source

For peer review only

For peer review only - http://bmjopen.bmj.com/site/about/guidelines.xhtml

Figure 1: Flow diagram of scholarly and public attention surrounding the DAPT study identified

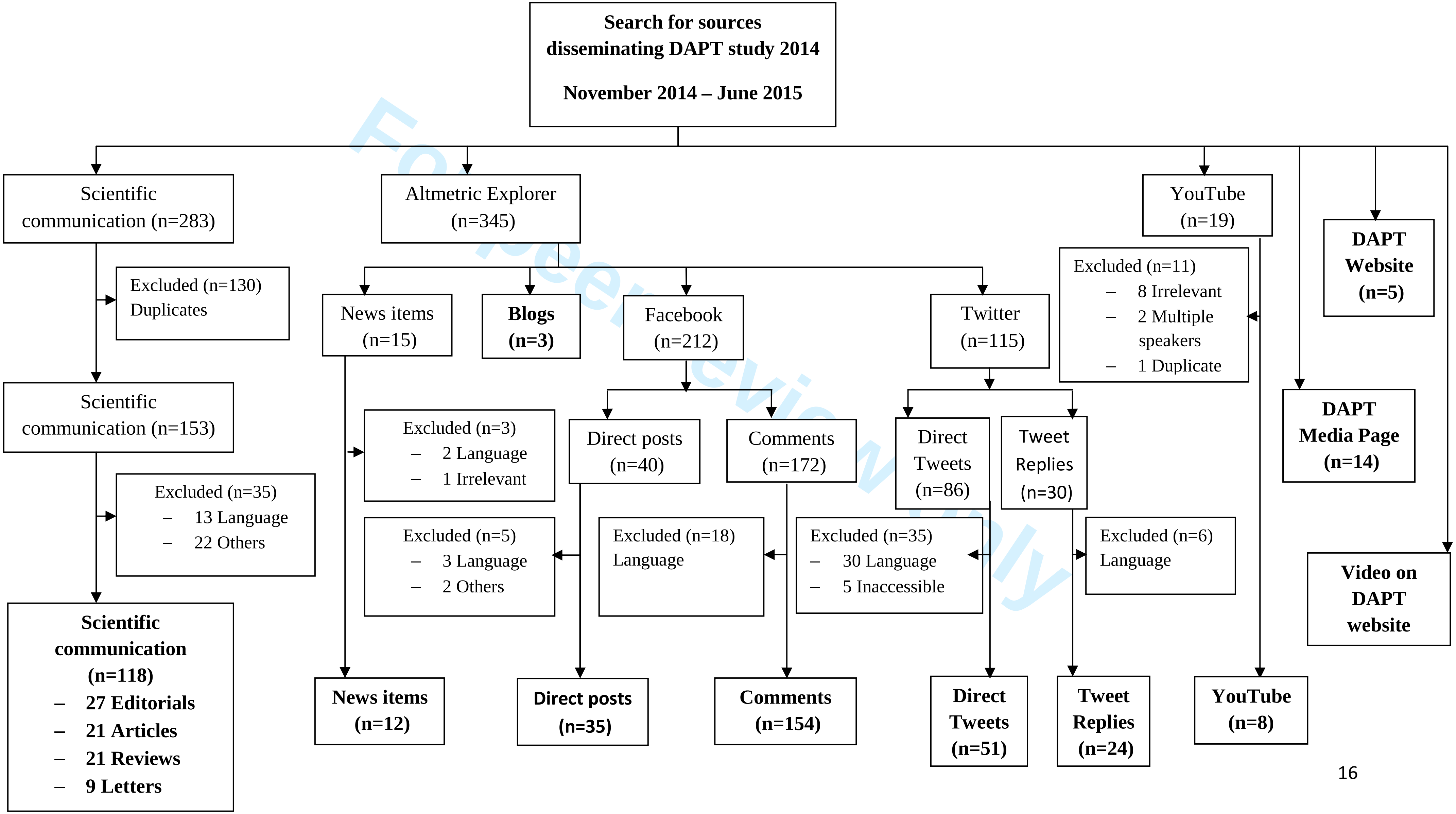

For peer review only - http://bmjopen.bmj.com/site/about/guidelines.xhtml

Figure 2: Content of the scholarly and public attention surrounding the DAPT study (n = 425)

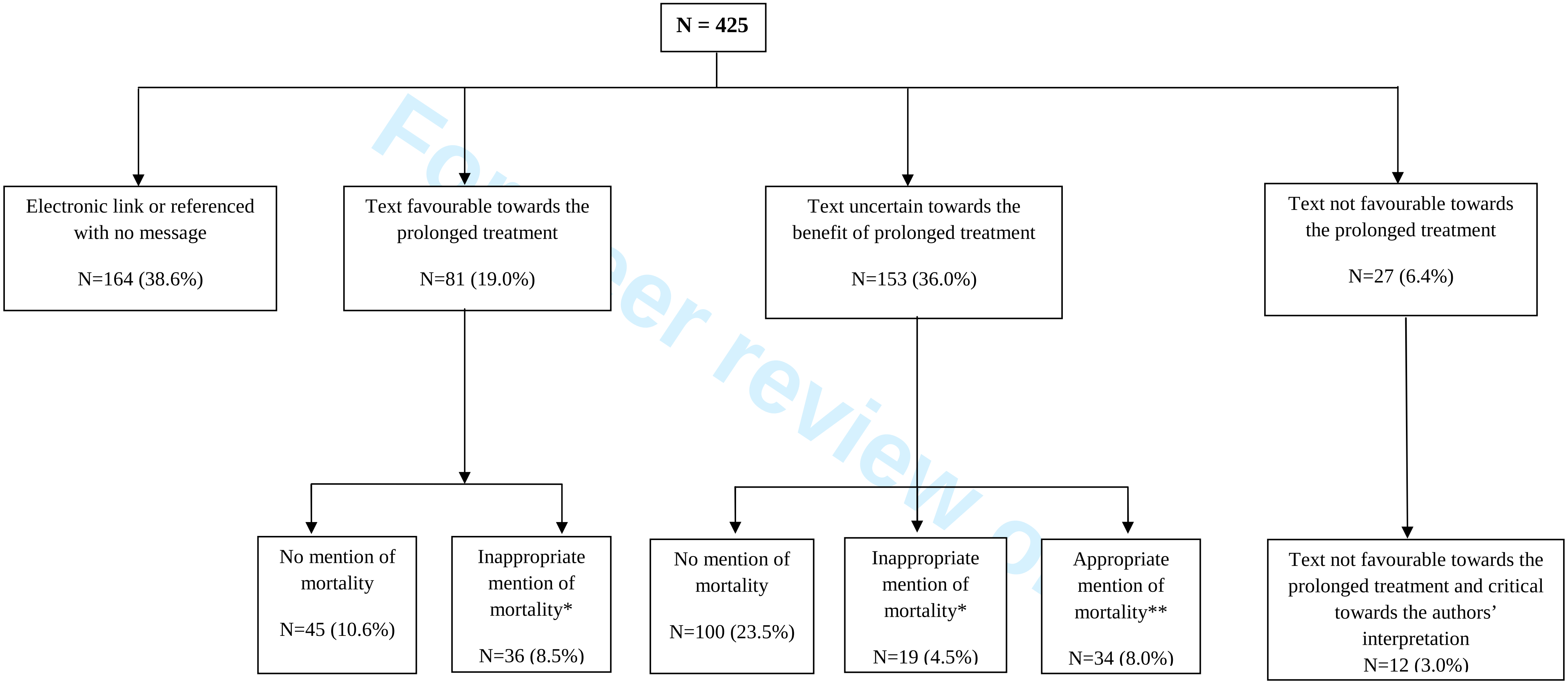

*Increased mortality reported with the authors' questionable explanation clearing the prolonged treatment responsibility in the increased risk of mortality

** Increased mortality reported without any explanation

For peer review only - http://bmjopen.bmj.com/site/about/guidelines.xhtml

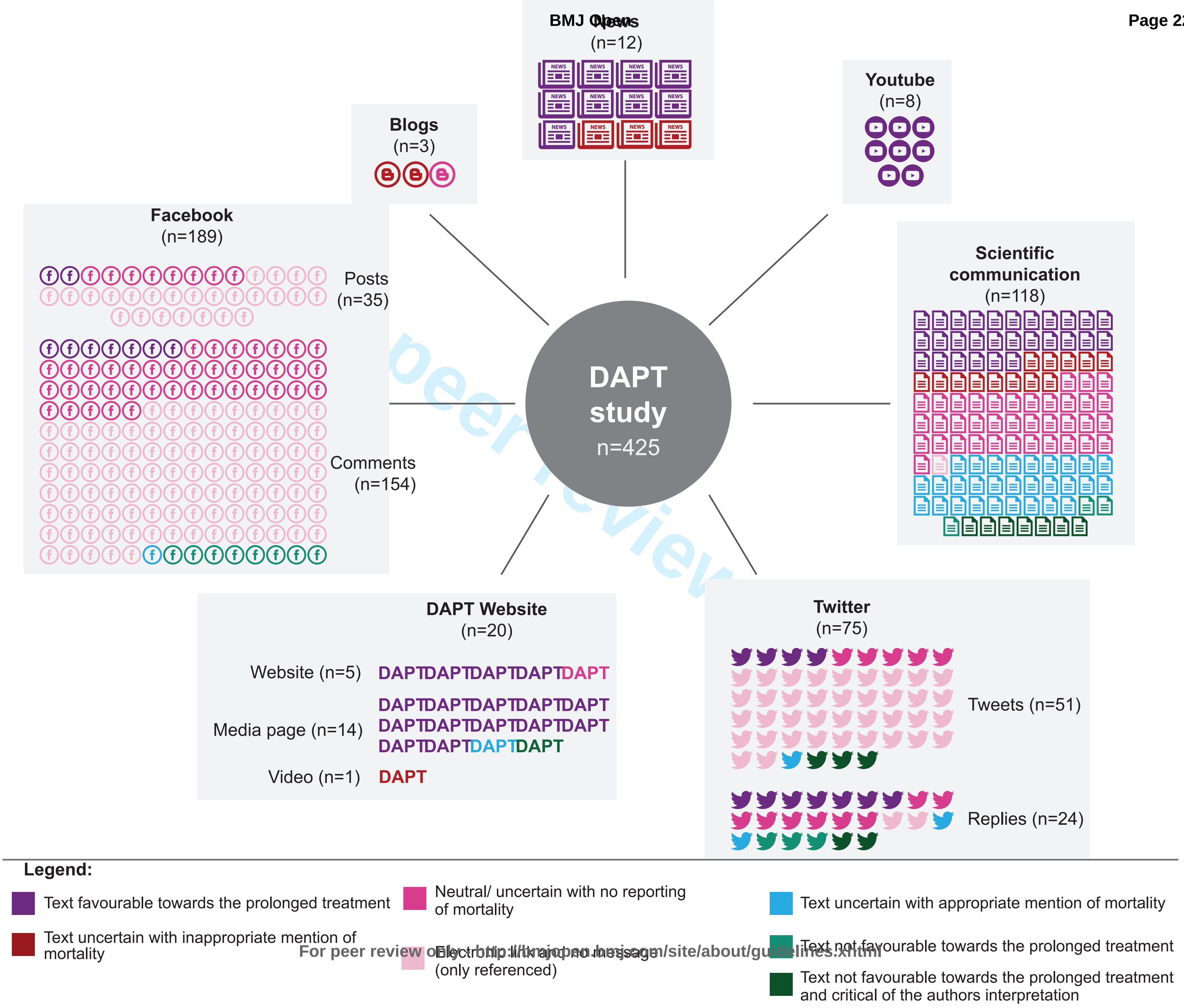
News
(n=12)
NEWS
Blogs
(n=3)
Youtube
(n=8)
Facebook
(n=189)
Posts
(n=35)
Comments
(n=154)
DAPT
study
n=425
Scientific
communication
(n=118)
DAPT Website
(n=20)
Website (n=5)
DAPTDAPTDAPTDAPTDAPT
Media page (n=14)
DAPTDAPTDAPTDAPTDAPT
DAPTDAPTDAPTDAPTDAPT
DAPTDAPTDAPTDAPT
Video (n=1)
DAPT
Twitter
(n=75)
Tweets (n=51)
Replies (n=24)
Legend:
Text favourable towards the prolonged treatment
Neutral/ uncertain with no reporting of mortality
Text uncertain with appropriate mention of mortality
Text uncertain with inappropriate mention of mortality
Electronic mail message (only referenced)
Text not favourable towards the prolonged treatment
Text not favourable towards the prolonged treatment and critical of the authors interpretation

S1 Table: Content of the scholarly and public attention surrounding the DAPT study by source (n = 425)

Category	Overall n=425	Scientific communication 118 (27.7)	News 12 (2.8)	Blogs 3 (0.7)	Facebook posts 189 (44.4)	Tweets 75 (17.6)	YouTube 8 (1.9)	DAPT Website 20 (4.7)
Text favourable about the prolonged treatment	81 (19.1)	28 (23.7)	9 (75.0)	-	9 (4.8)	11 (14.7)	(100)	16 (80.0)
Text uncertain, with inappropriate mention of mortality	19 (4.5)	13 (11.0)	3 (25.0)	2 (66.7)	-	-	-	1 (5.0)
Electronic link	151 (35.5)	-	-	-	113 (59.8)	38 (50.6)	-	-
Referenced with no message	13 (3.1)	1 (0.8)	-	-	10 (5.3)	2 (2.7)	-	-
Text uncertain, with no mention of mortality	100 (23.5)	37 (31.4)	-	1 (33.3)	48 (25.4)	13 (17.3)	-	1 (5.0)
Text uncertain, with appropriate mention of mortality	34 (8.0)	29 (24.6)	-	-	1 (0.5)	3 (4.0)	-	1 (5.0)
Text not favourable about the prolonged treatment	15 (3.5)	3 (2.5)	-	-	8 (4.2)	3 (4.0)	-	1 (5.0)
Text not favourable about the prolonged treatment and critical of the authors' interpretation	12 (3.0)	7 (6.0)	-	-	-	5 (6.7)	-	-

For peer review only - http://bmjopen.bmj.com/site/about/guidelines.xhtml

STROBE 2007 (v4) Statement—Checklist of items that should be included in reports of *cross-sectional studies*

Section/Topic	Item #	Recommendation	Reported on page #
**Title and abstract**	1	(*a*) Indicate the study's design with a commonly used term in the title or the abstract	1
		(*b*) Provide in the abstract an informative and balanced summary of what was done and what was found	2
**Introduction**			**4**
Background/rationale	2	Explain the scientific background and rationale for the investigation being reported	4, 5
Objectives	3	State specific objectives, including any prespecified hypotheses	5
**Methods**			**6**
Study design	4	Present key elements of study design early in the paper	6
Setting	5	Describe the setting, locations, and relevant dates, including periods of recruitment, exposure, follow-up, and data collection	6
Participants	6	(*a*) Give the eligibility criteria, and the sources and methods of selection of participants ***(no participants were involved in this study. Unit of study was the items disseminating DAPT study).***	7
Variables	7	Clearly define all outcomes, exposures, predictors, potential confounders, and effect modifiers. Give diagnostic criteria, if applicable	7
Data sources/ measurement	8*	For each variable of interest, give sources of data and details of methods of assessment (measurement). Describe comparability of assessment methods if there is more than one group	7, 8
Bias	9	Describe any efforts to address potential sources of bias	*No*
Study size	10	Explain how the study size was arrived at	6
Quantitative variables	11	Explain how quantitative variables were handled in the analyses. If applicable, describe which groupings were chosen and why	NA
Statistical methods	12	(*a*) Describe all statistical methods, including those used to control for confounding	8
		(*b*) Describe any methods used to examine subgroups and interactions	
		(*c*) Explain how missing data were addressed	
		(*d*) If applicable, describe analytical methods taking account of sampling strategy	
		(*e*) Describe any sensitivity analyses	
**Results**			9

For peer review only

Participants	13*	(a) Report numbers of individuals at each stage of study—eg numbers potentially eligible, examined for eligibility, confirmed eligible, included in the study, completing follow-up, and analysed (in flow diagram)	9, 17
		(b) Give reasons for non-participation at each stage (in flow diagram)	17
		(c) Consider use of a flow diagram	9
Descriptive data	14*	(a) Give characteristics of study participants (eg demographic, clinical, social) and information on exposures and potential confounders (Unit of study was the items disseminating DAPT study)	9
		(b) Indicate number of participants with missing data for each variable of interest	
Outcome data	15*	Report numbers of outcome events or summary measures	9, 10
Main results	16	(*a*) Give unadjusted estimates and, if applicable, confounder-adjusted estimates and their precision (eg, 95% confidence interval). Make clear which confounders were adjusted for and why they were included	9, 10
		(*b*) Report category boundaries when continuous variables were categorized	
		(*c*) If relevant, consider translating estimates of relative risk into absolute risk for a meaningful time period	
Other analyses	17	Report other analyses done—eg analyses of subgroups and interactions, and sensitivity analyses	NA
**Discussion**			11
Key results	18	Summarise key results with reference to study objectives	11
Limitations	19	Discuss limitations of the study, taking into account sources of potential bias or imprecision. Discuss both direction and magnitude of any potential bias	11, 12
Interpretation	20	Give a cautious overall interpretation of results considering objectives, limitations, multiplicity of analyses, results from similar studies, and other relevant evidence	11, 12
Generalisability	21	Discuss the generalisability (external validity) of the study results	11
**Other information**			12-14
Funding	22	Give the source of funding and the role of the funders for the present study and, if applicable, for the original study on which the present article is based	13

*Give information separately for cases and controls in case-control studies and, if applicable, for exposed and unexposed groups in cohort and cross-sectional studies.

Note: An Explanation and Elaboration article discusses each checklist item and gives methodological background and published examples of transparent reporting. The STROBE checklist is best used in conjunction with this article (freely available on the Web sites of PLoS Medicine at http://www.plosmedicine.org/, Annals of Internal Medicine at http://www.annals.org/, and Epidemiology at http://www.epidem.com/). Information on the STROBE Initiative is available at www.strobe-statement.org.

For peer review only

BMJ Open

Dissemination of 2014 Dual Anti-platelet Therapy (DAPT) trial results: A systematic review of scholarly and media attention over 7 months

Journal:	*BMJ Open*
Manuscript ID	bmjopen-2016-014503.R1
Article Type:	Research
Date Submitted by the Author:	30-Jun-2017
Complete List of Authors:	Sharp, Melissa K; Columbia University, Mailman School of Public Health; INSERM UMR 1153, Center of Research in Epidemiology and Statistics Sorbonne Paris Cité, Center of Epidemiology and Clinical Research<br>HANEEF, Romana; INSERM UMR 1153, Center of Research in Epidemiology and Statistics Sorbonne Paris Cité, Center of Epidemiology and Clinical Research; University of Paris Descartes<br>Ravaud, Philippe; INSERM UMR 1153, Center of Research in Epidemiology and Statistics Sorbonne Paris Cité, Center of Epidemiology and Clinical Research; University of Paris Descartes<br>Boutron, Isabelle; INSERM UMR 1153, Center of Research in Epidemiology and Statistics Sorbonne Paris Cité, Center of Epidemiology and Clinical Research; University of Paris Descartes
**Primary Subject Heading**:	Epidemiology
Secondary Subject Heading:	Qualitative research, Public health, Research methods, Communication
Keywords:	DAPT Therapy, Misleading interpretation, Mortality, Public attention, Critical opinion

SCHOLARONE™
Manuscripts

Dissemination of 2014 Dual Anti-platelet Therapy (DAPT) trial results: A systematic review of scholarly and media attention over 7 months

Melissa K Sharp• (1,2), Romana Haneef• (2,3,4), Philippe Ravaud (1,2,3,4,5), Isabelle Boutron*(2,3,4,5)

(1) Columbia University, Mailman School of Public Health, New York, NY, USA

(2) METHODS Team, Center of Research in Epidemiology and Statistics Sorbonne Paris Cité, UMR 1153, INSERM, Paris, France.

(3) University Paris Descartes, Sorbonne Paris Cité, Paris, France

(4) Centre d'Épidémiologie Clinique, AP-HP (Assistance Publique des Hôpitaux de Paris), Hôpital Hôtel Dieu, Paris, France

(5) Cochrane France, Paris, France

• These authors contributed equally to this work as a first author.

*Corresponding author:

Isabelle Boutron

Centre d'Epidémiologie Clinique

Hôpital Hôtel Dieu,

1, Place du parvis Notre Dame

75004 Paris Cedex 4

E-mail : isabelle.boutron@aphp.fr

Tel : 00 33 1 42 34 78 33

Fax : 00 33 1 42 34 87 90

For peer review only

For peer review only - http://bmjopen.bmj.com/site/about/guidelines.xhtml

Abstract

Objective: To explore how the results from the 2014 DAPT trial were disseminated to the scientific community and online media.

Design: A cross-sectional study of scholarly and public attention surrounding the DAPT study.

Settings: Data were collected from the ISI Web of Knowledge, Google Scholar, PubMed Commons, EurekAlert, the DAPT study website (www.daptstudy.org), and the *New England Journal of Medicine* website *(for scholarly attention)* and Altmetric Explorer, Snap Bird, YouTube *(for public attention)* citing DAPT study results appearing from November 16, 2014 to June 10, 2015.

Participants: No participants were involved in this study.

Main outcome measure: Proportion of contents highlighting the increased risk of mortality and critical to the author's questionable interpretation of the results.

Results: We identified 425 items reported by 7 sources; 164 (39%) disseminated the authors' questionable interpretation via an electronic link or a reference, with no additional text. Among 81 items (19%), the message favoured prolonged treatment and consequently overstated the article conclusions. Among 119 items (28%), the text was uncertain about the benefit of prolonged treatment but was reported with no or inappropriate mention of increased risk of mortality. Only 34 items (8%) were uncertain about the benefit of prolonged treatment and appropriately mentioned increased risk of mortality. In all, 27 items (6%) did not favour prolonged treatment, and only 12 of these (3%) clearly raised some concerns about the reporting of increased risk of death.

Conclusion: The amount of contents criticizing the interpretation of the DAPT study results was limited.

For peer review only

Strengths and limitation of this study

- Our method involved a broad search strategy, ensured to capture an extensive and representative sample of contents citing the 2014 DAPT trial for both scholarly and public attention.
- Our systematic approach to analyze the text of contents provides a comprehensive overview of dissemination of the study results.
- This study focused on only a specific trial publication and results are not generalizable to other studies.

For peer review only

For peer review only - http://bmjopen.bmj.com/site/about/guidelines.xhtml

INTRODUCTION

The development of optimal coronary stent replacement has progressed rapidly over recent years [1]. In the United States, almost 700,000 stents are placed every year and there is an increasing trend for its use in Europe [2]. Dual antiplatelet therapy (DAPT) (i.e., P2Y12-receptor inhibitor combined with aspirin) is recommended after placement of coronary stents to prevent thrombotic complications [3]. The optimal duration of DAPT has been debated [4-8].

In December 2014, the Harvard Clinical Research Institute (HCRI) released the results of the DAPT study, the largest international randomized controlled trial to date [9]. The trial aimed to determine the benefits and risks of continuing DAPT beyond 1 year after placement of a coronary stent [9]. A total of 9,961 adult patients were randomly assigned to continue thienopyridine treatment or to receive a placebo for 30 months. Continued therapy reduced the rate of stent thrombosis (0.4% vs.1.4%; p<0.001) and major adverse cardiovascular and cerebrovascular events (MACCEs) (2.1% vs. 4.1%; p<0.001), with an expected increase in the rate of moderate or severe bleeding (2.5% vs. 1.6%; p=0.001) [9]. However, continued therapy was also associated with an increase of 36% in all-cause mortality (2.0% vs. 1.5%; hazard ratio 1.36 [95% CI 1.00 to 1.85]; P=0.05).

The results of the DAPT study were published in the *New England Journal of Medicine* (*NEJM*) [9] after their presentation at the American Health Association Conference, in November 2014. However, the reporting of the results raised some concerns [10, 11]. Particularly, the abstract conclusions did not mention the increased risk of mortality. Furthermore, the discussion included questionable explanations based on post-hoc analyses to clear the role of prolonged thienopyridine treatment in this increased risk of mortality. For this purpose, the authors had split the analysis by cause of death, which was not powered to show a statistically significant difference. They focused on the increase in cancer-related

For peer review only - http://bmjopen.bmj.com/site/about/guidelines.xhtml

death (0.62% vs 0.28%, p = 0.02). The results were interpreted as being related to an imbalance at baseline in patients with a history of cancer before enrolment (9.8% vs 9.5%). To confirm, the authors performed a post-hoc analysis excluding all deaths that could be related to cancer diagnosed before enrolment. Consequently, the results became statistically non-significant (0.50% vs 0.28%, p=0.11). This post-hoc exclusion of patients with an event is a concern.

We aimed to explore how the distorted interpretation of results from the DAPT trial was disseminated to the scientific community and online media and to assess whether this interpretation was criticized or not.

For peer review only

METHODS

We performed a cross-sectional study of scholarly and public attention surrounding the DAPT study.

Identification of scholarly and public attention surrounding the DAPT study

Scholarly attention

On June 2015, we searched the following electronic databases to identify responses to the DAPT study: ISI Web of Knowledge, Google Scholar, and PubMed Commons. We also searched the comments and citing articles on the *NEJM* website for the original article [9].

Public attention

We searched Altmetric Explorer [12-15] to identify all online attention (news, blogs, Twitter, Facebook, Google+, Mendeley, CiteULike) given to the DAPT study. Each identified social media source was then systematically evaluated to determine whether other posts were not captured by Altmetric Explorer. In addition, each original tweet was reviewed to find retweets, replies and favourites. Since Altmetric.com captures only tweets attached to the DOI (Digital Object Identifier) of the original DAPT article, we also used snapbird.org, a search engine that can search an individual Twitter account by using the *NEJM*'s Twitter account and the search terms "DAPT" and "dual antiplatelet therapy". We also searched EurekAlert! (a free online database for science press releases, www.eurekalert.org) for press releases dedicated to the DAPT study; YouTube (search terms "DAPT" and "dual antiplatelet therapy"); and pages dedicated to patients, clinicians and media at the DAPT study website (http://www.daptstudy.org).

For peer review only - http://bmjopen.bmj.com/site/about/guidelines.xhtml

Eligibility criteria

Two researchers (MS, RH) screened all items retrieved and selected all English-language items that cited the DAPT study and were released from November 16, 2014 to June 10, 2015. Any disagreements were resolved by discussion to reach consensus.

Content of scholarly and public attention surrounding the DAPT study

Two researchers (MS, RH) read the items from each source independently and evaluated them by using a preliminarily tested extraction form. Disagreements were resolved by discussion to reach consensus. If needed, a third researcher (IB) appraised the content.

We determined whether the source consisted of a reference or a link to the *NEJM* article reporting the DAPT study only or was a text commenting on the DAPT study. For a text commenting on the DAPT study, we checked whether the original study authors were involved in writing the text or not. Our main outcome of interest was the proportion of contents highlighting the increased risk of mortality and critical to the author's questionable interpretation of the results. We determined whether

- the primary efficacy outcomes (i.e., stent thrombosis and MACCE) were reported
- the safety outcomes related to moderate or severe bleeding were reported
- the increased risk of mortality with prolonged treatment was reported
- the authors' questionable explanation clearing the responsibility of prolonged treatment in the increased risk of mortality was reported or criticized
- the content of the text was 1) favouring the prolonged treatment and consequently overstating the article conclusion, 2) uncertain about the benefit of the prolonged treatment (i.e., statement of both the beneficial effect, and increased risk of bleeding, text ending with a question mark, use of "may or might" or reporting that the study needs further research), or 3) not favouring the prolonged treatment [16].

For peer review only

Overall, we classified the sources based on the text of contents as follows:

1. Text favouring the prolonged treatment
2. Text uncertain (about the benefit of prolonged treatment) with inappropriate mention of mortality
3. Text neutral/uncertain (about the benefit of prolonged treatment) with no mention of mortality
4. Electronic link or referenced with no message
5. Text uncertain (about the benefit of prolonged treatment) with appropriate mention of mortality
6. Text not favouring the prolonged treatment
7. Text not favouring the prolonged treatment and critical of the authors' interpretation

Statistical analysis

We calculated frequencies and percentages (%) for qualitative variables and median (interquartile range) for quantitative variables.

For peer review only - http://bmjopen.bmj.com/site/about/guidelines.xhtml

RESULTS

Identification of scholarly and public attention surrounding the DAPT study

From all sources, we selected and appraised 425 items: 118 scientific communications, 12 news items, 3 blogs, 189 Facebook posts or comments, 75 tweets or replies, 8 videos on YouTube, 14 DAPT media pages, 5 DAPT website pages and 1 video on the DAPT website (Figure 1). The original study authors were directly involved in 35 items. Details of 118 scientific communications are in *Appendix 1*.

Reporting of the content

The texts of contents are described in Figure 2 (overall) and Figure 3 (by source). Overall, 164 items (39%) involved disseminating the authors' questionable reporting and interpretation via an electronic link (n=151, 36%) or reference (n=13; 3%), with no additional text or message. Among 81 items (19%), the message favoured the prolonged treatment and therefore overstated the article conclusions. For example, the DAPT study website dedicated to patients reported that "*It is important that patients who currently take a thienopyridine anti-clotting medication (clopidogrel or prasugrel) do not stop taking their medication. […] The benefits of continuing dual antiplatelet therapy for one year, according to current guidelines, far outweigh the risks*." Among 153 items (36%), the text was uncertain about the benefit of prolonged treatment but was reported with no mention of the increased risk of mortality (n=100, 24%) or the questionable explanation clearing the responsibility of prolonged treatment (n=19; 4.5%). Overall, 34 items (8%) were uncertain about the benefit of prolonged treatment but mentioned the increased risk of mortality. Only 27 (6%) did not favour prolonged treatment and only 12 of these (3%) clearly raised some concerns about the reporting of the increased risk of death. Further information on items by source is in *Appendix 2*.

For peer review only - http://bmjopen.bmj.com/site/about/guidelines.xhtml

Overall, 136 items (32%) reported efficacy outcomes (i.e., stent thrombosis and MACCEs), 127 (30%) safety outcomes and 113 (27%) both efficacy and safety outcomes.

A total of 100 items (24%) did not mention mortality, but when mortality was mentioned, in 19 items (5%), it was reported with the authors' questionable justification for prolonged treatment.

For peer review only

For peer review only - http://bmjopen.bmj.com/site/about/guidelines.xhtml

DISCUSSION

We describe the dissemination of the 2014 DAPT study findings in scientific community and to the public via different sources such as news, blogs, and social media. Our assessment of 425 items disseminating the DAPT study results showed that only 8% of the items mentioned some uncertainty about the benefit of prolonged treatment and included an appropriate mention of the increased risk of mortality. Furthermore, only 12 items (3%) clearly raised some concerns about the reporting of the increased risk of death. This study adds to the burgeoning literature on the biased dissemination of research results. Previous studies have focused on publication bias [17], selective reporting of outcomes [17-22], and spin [19, 23, 24].

However, this is the first study to our knowledge to focus on both scholarly and public dissemination of study results. Our study highlighted an unmet need of scientific communication in the media, whose importance in dissemination of scientific data is becoming increasingly relevant. These findings could be helpful for the entire community for better understanding how scientific knowledge is disseminated.

Our approach involved a broad search strategy and multiple search engines, which ensured the capture of an extensive and representative sample of contents discussing the DAPT study results. Each social media item from Altmetric was systematically reviewed for additional content that may have been missed, and several different search engines were used. We captured items that were published over the course of many months, which highlighted the perpetuation and continuation of the dissemination of the questionable interpretations. The inclusion period for sources seemed to be more than sufficient because tweets linked to scientific articles have been shown to taper off well before our cut-off point (7 months) [25]. In addition, 2 independent researchers assessed each source by using a standardized data extraction form and disagreements were resolved by consensus.

For peer review only - http://bmjopen.bmj.com/site/about/guidelines.xhtml

However, our study has some limitations. First, this study focused on only a specific trial publication and results are not generalizable to other studies. However, the article we focused on was among the top 5 of all research outputs and within the 99th percentile of articles on Altmetric. Second, the data extraction involved some subjectivity; however, we tried to address this by using a standardized data extraction form and independent assessment as well as consensus among 2 researchers. Third, despite our best efforts, we cannot ensure that our search strategy was all-encompassing because of the breadth of social media. Finally, we did not explore the balance between efficacy and safety outcomes with DAPT treatment.

Our aim was not to resolve the controversy about DAPT duration and this debate is still ongoing. The OPITUDAL trial did not find an increased risk of death with the prolonged treatment; on the contrary, the risk of death was lower with the prolonged treatment [26]. Several meta-analyses found conflicting results [4, 5, 8, 27, 28]. The researchers involved in the DAPT trial concluded in a meta-analysis published in *The Lancet* that prolonged DAPT duration was not associated with a difference in risk of all-cause mortality [29]. Three meta-analyses, published later by different teams, showed prolonged DAPT associated with increased risk of all-cause mortality [4, 5, 8]. More recently, other meta-analyses did not find a statistically significant increase in all-cause mortality [27, 28]. Most of these meta-analyses warranted further research with extended DAPT.

However, these results are difficult to interpret because of different definitions of short (1, 3, 6, or 12 months) and extended (6, 12, 24 or > 24 months) durations, which varied across studies. Furthermore, different durations of follow-up and types of stents could also influence the results.

CONCLUSIONS

For peer review only - http://bmjopen.bmj.com/site/about/guidelines.xhtml

Dissemination of the DAPT study results to the scientific community and on different media sources rarely criticized the interpretation of the study results.

Supplementary Data

Appendix 1: Detail of 118 scientific communications

Appendix 2: Content of scholarly and public attention surrounding the DAPT study by source.

For peer review only

For peer review only - http://bmjopen.bmj.com/site/about/guidelines.xhtml

Acknowledgements

We thank Elise Diard for help in creating Figure 2. We acknowledge support from Altmetric for free access to “Altmetric Explorer”. We acknowledge the assistance in English language proofreading by Laura Smales (BioMedEditing, Toronto, Canada).

Isabelle Boutron and Philippe Ravaud submitted a letter to the *NEJM* following the publication of the DAPT study to highlight the inadequate reporting in the abstract conclusions, but the letter was rejected.

Contributors

Study conception, design, selection of contents and data extraction: MS, RH. Study conception and design: MS, RH, IB. Selection of contents, data extraction: MS, RH. Analysis of data and interpretation of results: RH, PR, IB. Contributed to the writing of the manuscript: MS, RH, PR, IB. All authors read and approved the final manuscript.

Funding Source

This study did not receive any funding.

Competing interests

None declared.

Ethical approval

Not needed

For peer review only

Data sharing

All relevant data are included in this manuscript. Details of text content are available upon request for academic researchers.

Open Access

This is an Open Access article distributed in accordance with the Creative Commons Attribution Non Commercial (CC BY-NC 4.0) license, which permits others to distribute, remix, adapt, build upon this work non-commercially, and license their derivative works on different terms, provided the original work is properly cited and the use is non-commercial. See: http://creativecommons.org/licenses/by-nc/4.0/.

For peer review only

For peer review only - http://bmjopen.bmj.com/site/about/guidelines.xhtml

References

1. Wilson WM. Advances in coronary stent technology: current expectations and new developments. *Research Reports in Clinical Cardiology* 2013.
2. Mozaffarian D, Benjamin EJ, Go AS, et al. Heart disease and stroke statistics--2015 update: a report from the American Heart Association. *Circulation* 2015; **131**: e29-322.
3. Palmerini T, Dangas G, Mehran R, et al. Predictors and Implications of Stent Thrombosis in Non–ST-Segment Elevation Acute Coronary Syndromes: The ACUITY Trial. *Circulation: Cardiovascular Interventions* 2011; **4**: 577-84.
4. Palmerini T, Benedetto U, Bacchi-Reggiani L, et al. Mortality in patients treated with extended duration dual antiplatelet therapy after drug-eluting stent implantation: a pairwise and Bayesian network meta-analysis of randomised trials. *The Lancet* 2015.
5. Spencer FA, Prasad M, Vandvik PO, Chetan D, Zhou Q, Guyatt G. Longer- Versus Shorter-Duration Dual-Antiplatelet Therapy After Drug-Eluting Stent PlacementA Systematic Review and Meta-analysisDuration of Dual-Antiplatelet Therapy After Drug-Eluting Stents. *Annals of Internal Medicine* 2015; **163**: 118-26.
6. Levine GN, Bates ER, Blankenship JC, et al. 2011 ACCF/AHA/SCAI Guideline for Percutaneous Coronary InterventionA Report of the American College of Cardiology Foundation/American Heart Association Task Force on Practice Guidelines and the Society for Cardiovascular Angiography and Interventions. *Journal of the American College of Cardiology* 2011; **58**: e44-e122.
7. Windecker S, Kolh P, Alfonso F, et al. 2014 ESC/EACTS Guidelines on myocardial revascularization. *The Task Force on Myocardial Revascularization of the European Society of Cardiology (ESC) and the European Association for Cardio-Thoracic Surgery (EACTS)Developed with the special contribution of the European Association of Percutaneous Cardiovascular …* 2014; **35**: 2541-619.
8. Navarese EP, Andreotti F, Schulze V, et al. Optimal duration of dual antiplatelet therapy after percutaneous coronary intervention with drug eluting stents: meta-analysis of randomised controlled trials. *British Medical Journal* 2015; **350**.
9. Mauri L, Kereiakes DJ, Yeh RW, et al. Twelve or 30 Months of Dual Antiplatelet Therapy after Drug-Eluting Stents. *New England Journal of Medicine* 2014; **371**: 2155-66.
10. Lipkin D. 1 out of a hundred patient will benefit from extended dual Rx. 2014.
11. Gupta R. Dual antiplatelets :Walking on a tight rope. 2014.
12. Altmetric, https://www.altmetric.com/whatwedo.php. 2012.
13. Haustein S, Costas R, Larivière V. Characterizing Social Media Metrics of Scholarly Papers: The Effect of Document Properties and Collaboration Patterns. *PLoS ONE* 2015; **10**: e0120495.
14. Liu CL, Xu YQ, Wu H, Chen SS, Guo JJ. Correlation and Interaction Visualization of Altmetric Indicators Extracted From Scholarly Social Network Activities: Dimensions and Structure. *Journal of Medical Internet Research* 2013; **15**: e259.
15. Thelwall M, Haustein S, Larivière V, Sugimoto CR. Do Altmetrics Work? Twitter and Ten Other Social Web Services. *PLoS ONE* 2013; **8**: e64841.
16. Yank V, Rennie D, Bero LA. Financial ties and concordance between results and conclusions in meta-analyses: retrospective cohort study. *BMJ* 2007; **335**: 1202-5.
17. Dwan K, Altman DG, Arnaiz JA, et al. Systematic Review of the Empirical Evidence of Study Publication Bias and Outcome Reporting Bias. *PLoS ONE* 2008; **3**: e3081.
18. Riveros C, Dechartres A, Perrodeau E, Haneef R, Boutron I, Ravaud P. Timing and Completeness of Trial Results Posted at ClinicalTrials.gov and Published in Journals. *PLoS Med* 2013; **10**: e1001566.
19. Boutron I, Dutton S, Ravaud P, Altman DG. Reporting and interpretation of randomized controlled trials with statistically nonsignificant results for primary outcomes. *JAMA* 2010; **303**.

For peer review only

For peer review only - http://bmjopen.bmj.com/site/about/guidelines.xhtml

20. Tang E, Ravaud P, Riveros C, Perrodeau E, Dechartres A. Comparison of serious adverse events posted at ClinicalTrials.gov and published in corresponding journal articles. *BMC Medicine* 2015; **13**: 1-8.
21. Mathieu S, Boutron I, Moher D, Altman DG, Ravaud P. Comparison of registered and published primary outcomes in randomized controlled trials. *JAMA* 2009; **302**.
22. Chan A, Hróbjartsson A, Haahr MT, Gøtzsche PC, Altman DG. Empirical evidence for selective reporting of outcomes in randomized trials: Comparison of protocols to published articles. *JAMA* 2004; **291**: 2457-65.
23. Lazarus C, Haneef R, Ravaud P, Boutron I. Classification and prevalence of spin in abstracts of non-randomized studies evaluating an intervention. *BMC Medical Research Methodology* 2015; **15**: 1-8.
24. Yavchitz A, Boutron I, Bafeta A, et al. Misrepresentation of Randomized Controlled Trials in Press Releases and News Coverage: A Cohort Study. *PLoS Med* 2012; **9**: e1001308.
25. Eysenbach G. Can Tweets Predict Citations? Metrics of Social Impact Based on Twitter and Correlation with Traditional Metrics of Scientific Impact. *J Med Internet Res* 2011; **13**: e123.
26. Helft G, Steg PG, Le Feuvre C, et al. Stopping or continuing clopidogrel 12 months after drug-eluting stent placement: the OPTIDUAL randomized trial. *European Heart Journal* 2016; **37**: 365-74.
27. Basaraba JE, Barry AR. Short- versus standard-term dual antiplatelet therapy after percutaneous coronary intervention with drug-eluting stent implantation: A meta-analysis. *J Cardiol* 2017; **69**: 353-8.
28. D'Ascenzo F, Moretti C, Bianco M, et al. Meta-Analysis of the Duration of Dual Antiplatelet Therapy in Patients Treated With Second-Generation Drug-Eluting Stents. *The American journal of cardiology* 2016; **117**: 1714-23.
29. Elmariah S, Mauri L, Doros G, et al. Extended duration dual antiplatelet therapy and mortality: a systematic review and meta-analysis. *The Lancet* 2015; **385**: 792-8.

For peer review only

Figure 1: Flow diagram of identified scholarly and public attention surrounding the DAPT study

For peer review only

For peer review only - http://bmjopen.bmj.com/site/about/guidelines.xhtml

Figure 2: Content of scholarly and public attention surrounding the DAPT study (n = 425)

For peer review only

*Increased risk of mortality reported with the authors' questionable explanation clearing the responsibility of prolonged treatment in the increased risk

** Increased risk of mortality reported without any explanation

For peer review only - http://bmjopen.bmj.com/site/about/guidelines.xhtml

Figure 3: Content of scholarly and public attention surrounding the DAPT study by source (n = 425)

For peer review only

For peer review only - http://bmjopen.bmj.com/site/about/guidelines.xhtml

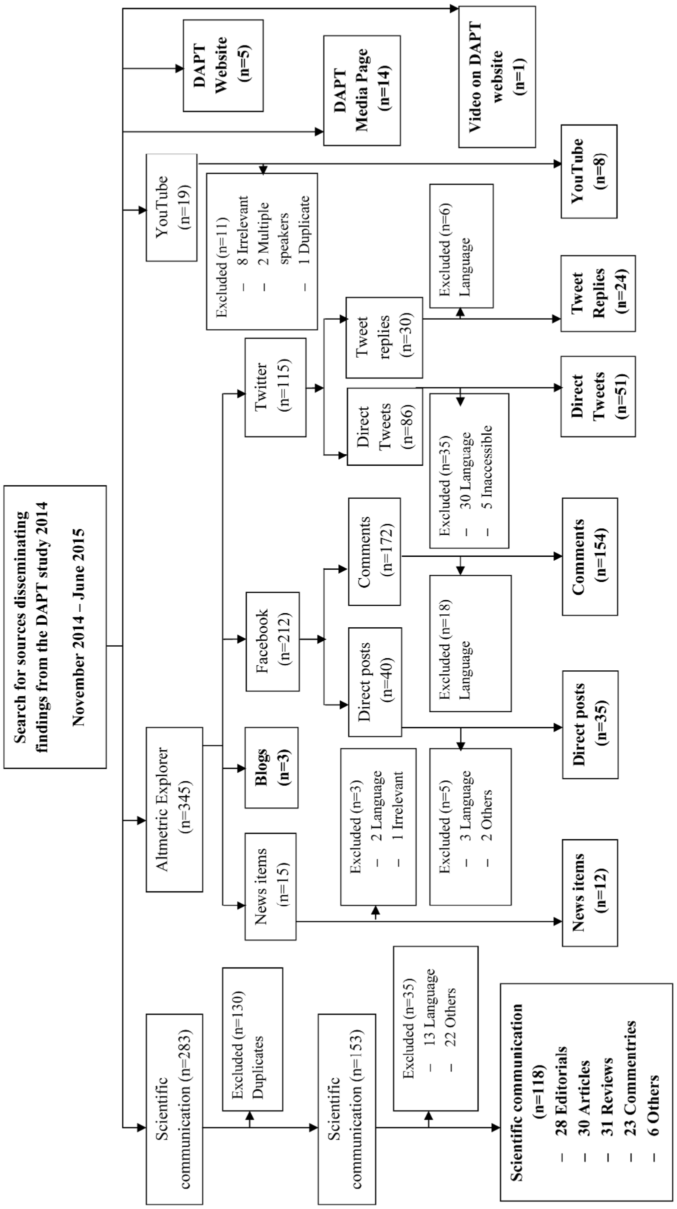

Flow diagram of identified scholarly and public attention surrounding the DAPT study

282x505mm (300 x 300 DPI)

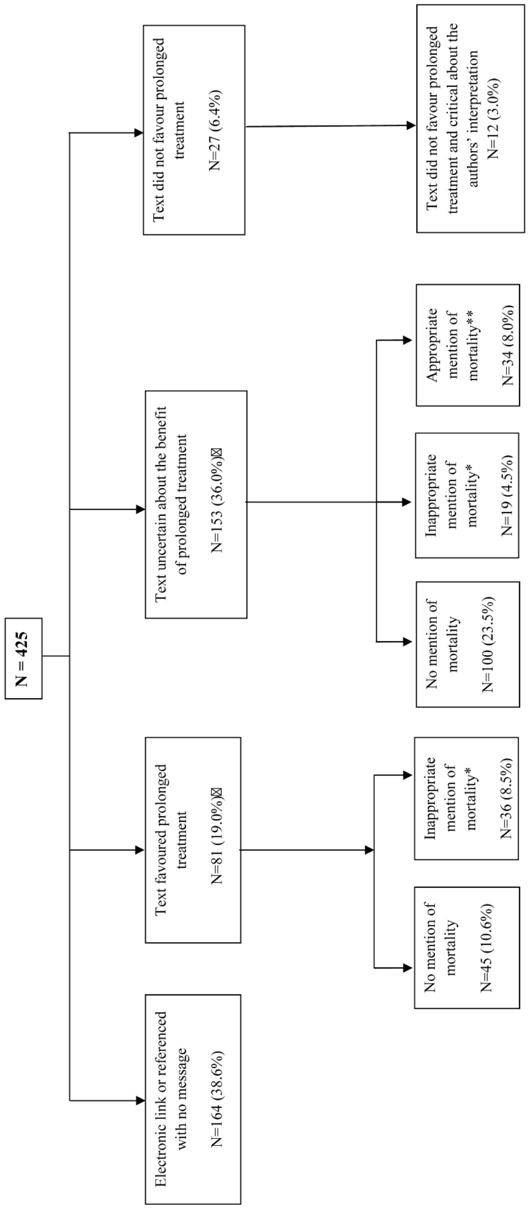

Content of the scholarly and public attention surrounding the DAPT study (n = 425)

119x276mm (300 x 300 DPI)

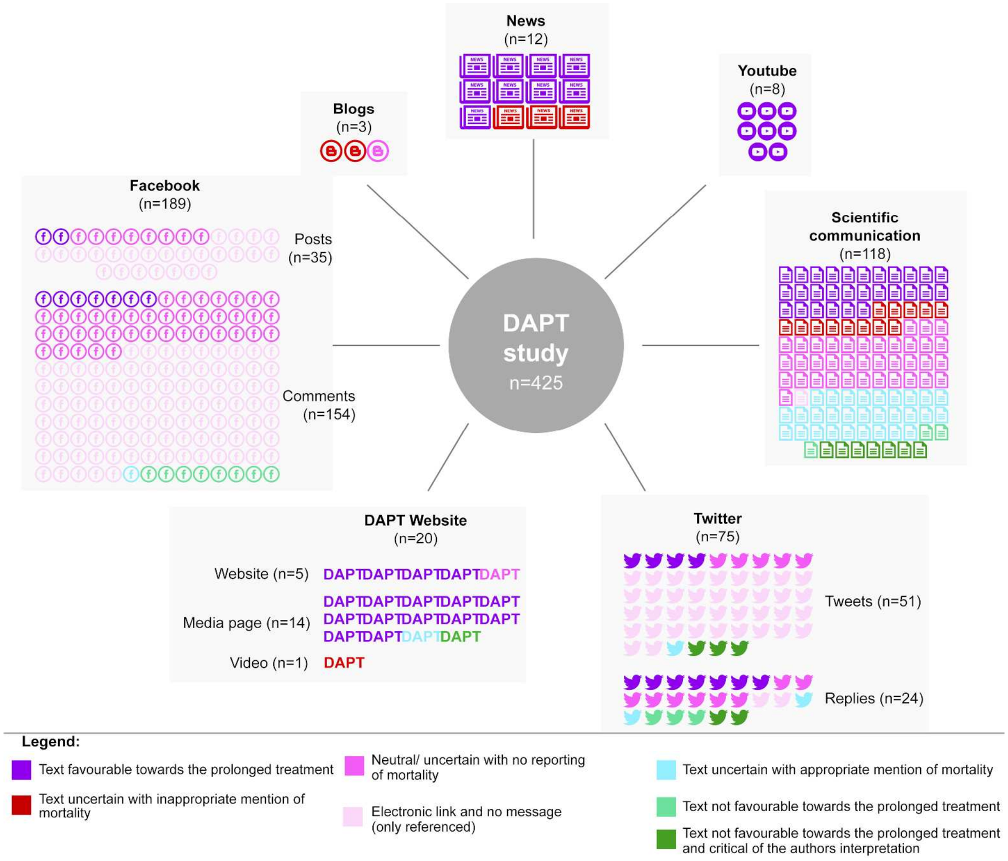

Content of the scholarly and public attention surrounding the DAPT study by source (n = 425)

256x219mm (300 x 300 DPI)

Appendix 1: Detail of 118 scientific communications					
**S/No**	**First Author**	**Year**	**Title**	**Journal**	**Type of scientific contribution**
1	Abo-salem	2015	Optimal duration of dual antiplatelet therapy after drug eluting stents: Meta-analysis of randomized trials	Cardiovascular Therapeutics	Article
2	Alfredsson	2015	Balancing the risks and benefits of long-term antiplatelet therapies for cardiovascular disease: clinical, research, and regulatory implications	J Am Heart Association	Editorial
3	AlJaroudi	2014	Review of Cardiovascular Literature	Journal of nuclear cardiology	Review
4	Angoulvant	2015	Dual antiplatelet therapy after acute coronary syndrome: a cardiologist-based optimal decision	Heart	Editorial
5	Aradi	2015	ATLANTIC: another reason to investigate the disconnect between stent thrombosis and mortality?	Thromosis & Haemostatis	Editorial
6	Auer	2015	Dual antiplatelet therapy duration and mortality	Lancet	Commentary
7	Becker	2015	Are at Least 12 Months of Dual Antiplatelet Therapy Needed for All Patients With Drug-Eluting Stents? Not All Patients With Drug-Eluting Stents Need at Least 12 Months of Dual Antiplatelet Therapy	Circulation	Editorial
8	Binder	2015	Duration of dual antiplatelet therapy after coronary artery stenting: where is the sweet spot between ischaemia and bleeding?	Europeaon Heart Journal	Editorial
9	Biondi-Zoccai	2015	Noncompliance and Cessation of Dual Antiplatelet Therapy After Coronary Stenting Looking at the Speck Rather Than Noticing the Log?	JACC- Cardiovascular Interventions	Editorial
10	Bonaca	2015	Long-term use of ticagrelor in patients with prior myocardial infarction	NEJM	Article
11	Brener	2015	Are at Least 12 Months of Dual Antiplatelet Therapy Needed for All Patients With Drug-Eluting Stents? All Patients With Drug-Eluting Stents Need at Least 12 Months of Dual Antiplatelet Therapy	Circulation	Editorial
12	Byrne	2015	Bioresorbable Drug-Eluting Stents: An Immature Technology in Need of Mature Application	JACC: Cardiovascular Interventions	Editorial

For peer review only - http://bmjopen.bmj.com/site/about/guidelines.xhtml

13	Capodanno	2015	What about the risk of thrombosis with bioresorbable scaffolds?	Eurointervention	Review
14	Capodanno	2015	Triple antithrombotic therapy in atrial fibrillation patients with acute coronary syndromes or undergoing percutaneous coronary intervention or transcatheter aortic valve replacement	Eurointervention	Editorial
15	Capodanno	2015	Impact of bridging with perioperative low-molecular-weight heparin on cardiac and bleeding outcomes of stented patients undergoing non-cardiac surgery	Thrombosis and Haemostasis	Article
16	Cassese	2015	Prolonged dual antiplatelet therapy after drug-eluting stenting: meta-analysis of randomized trials	Clinical Research in Cardiology	Article
17	Chow	2015	Drug-coated balloons: a novel advance in the percutaneous treatment of coronary and peripheral artery disease	Interventional Cardiology	Review
18	Cohen	2015	Long-term outcomes in high-risk patients with non-ST-segment elevation myocardial infarction	Journal of thrombosis and thrombolysis	Review
19	Collet	2015	Dual antiplatelet treatment after stenting–Authors' reply	The Lancet	Commentary
20	Colombo	2014	Dual Antiplatelet Therapy after Drug-Eluting Stents — How Long to Treat?	NEJM	Editorial
21	Cortese	2015	Drug-Coated Balloon angioplasty: an intriguing alternative for the treatment of Coronary Chronic Total Occlusions	International journal of cardiology	Letter
22	Costa	2015	Perspectives on the 2014 ESC/EACTS Guidelines on Myocardial Revascularization	Journal of cardiovascular translational research	Review
23	Costa	2015	Impact of clinical presentation on ischaemic and bleeding outcomes in patients receiving 6-or 24-month duration of dual-antiplatelet therapy after stent implantation: a pre-specified analysis from the PRODIGY (Prolonging Dual-Antiplatelet Treatment After Grading Stent-Induced Intimal Hyperplasia) trial	European Heart Journal	Article
24	Crea	2015	Sex differences in mechanisms, presentation and management of ischaemic heart disease	Atherosclerosis	Review
25	Cutlip	2014	Antiplatelet therapy after coronary artery stenting	UpToDate, Waltham, MA	Review

For peer review only

26	Curzen	2015	Prolonged antiplatelet therapy after drug-eluting stents	The Lancet	Commentary
27	de la Torre Hernandez	2015	Dual Antiplatelet Therapy for 6 Months vs 12 Months After New-generation Drug-eluting Stent Implantation: Matched Analysis of ESTROFA-DAPT and ESTROFA-2	Revista Española de Cardiología (English Edition)	Article
28	De Rango	2015	Dual Antiplatelet Therapy after Carotid Stenting: Lessons from'Big Brother'	European journal of vascular and endovascular surgery: the official journal of the European Society for Vascular Surgery	Editorial
29	Dhall	2014	Truth Vs hype	NEJM	Commentary
30	Dohan	2015	Duration of Dual Antiplatelet Therapy after Drug-Eluting Stents	NEJM	Commentary
31	Eisen	2015	Antiplatelet therapy: Defining the optimal duration of DAPT after PCI with DES	Nat Rev Cardiol	Others
32	Elmariah	2015	Extended duration dual antiplatelet therapy and mortality: a systematic review and meta-analysis	The Lancet	Article
33	Fanari	2015	Cost Effectiveness of Antiplatelet and Antithrombotic Therapy in The Setting of Acute Coronary Syndrome: current perspective and literature review	American Journal of Cardiovascular Drugs	Review
34	Fareed	2015	Antithrombotic therapy in 2014: Making headway in anticoagulant and antiplatelet therapy	Nature Reviews Cardiology	Review
35	Fiedler	2015	Duration of Triple Therapy in Patients Requiring Oral Anticoagulation After Drug-Eluting Stent Implantation The ISAR-TRIPLE Trial	Journal of the American College of Cardiology	Article
36	Genereux	2015	Stent Thrombosis and Dual Antiplatelet Therapy Interruption With Everolimus-Eluting Stents Insights From the Xience V Coronary Stent System Trials	Circulation: Cardiovascular Interventions	Article
37	Gilard	2015	Double Antiplatelet Therapy Duration: Standardize or Personalize?	Journal of the American College of Cardiology	Editorial

For peer review only

38	Gilchrist	2015	Vignettes of DES Failure	Catheterization and Cardiovascular Interventions	Editorial
39	Giustino	2015	Duration of Dual Antiplatelet Therapy After Drug-Eluting Stent Implantation: A Systematic Review and Meta-Analysis of Randomized Controlled Trials	Journal of the American College of Cardiology	Article
40	Gupta	2014	Balancing ischemia vs. bleeding-- Jury still out.	NEJM	Commentary
41	Gupta	2014	Dual antiplatelets :Walking on a tight rope	NEJM	Commentary
42	Habib	2015	Endothelialization of drug eluting stents and its impact on dual anti-platelet therapy duration	Pharmacol Res	Review
43	Henderson		Primecuts–This Week In The Journals	Clinical Correlations	Others
44	Hernandez	2015	2014 Update on Interventional Cardiology	Revista Española de Cardiología	Review
45	Huang	2015	Is the Duration of Dual Antiplatelet Therapy after Implantation of Drug-Eluting Stents the Longer the Better	Medical Principles and Practice	Letter
46	Husted	2015	Antithrombotic therapy for long-term secondary prevention of acute coronary syndrome in high-risk patients	Therapeutics and clinical risk management	Review
47	Huynh	2015	Antiplatelet therapy: Risks and benefits of extended DAPT after stenting	Nat Rev Cardiol	Others
48	Iqbal	2015	The year in cardiology 2014: coronary intervention	European Heart Journal	Review
49	Kumana	2015	Absolute benefits and harms of dual antiplatelet therapy after drug eluting stenting	Hong Kong Medical Journal	Article
50	Keaney	2015	Balancing the Risks and Benefits of Dual Platelet Inhibition	NEJM	Editorial

For peer review only

51	Kereiakes	2015	Efficacy and Safety of a Novel Bioabsorbable Polymer-Coated, Everolimus-Eluting Coronary Stent The EVOLVE II Randomized Trial	Circulation-Cardiovascular Interventions	Article
52	Kereiakes	2015	Antiplatelet therapy duration following bare metal or drug-eluting coronary stents: The dual antiplatelet therapy randomized clinical trial	JAMA	Article
53	Kirtane	2015	Should all stent patients have prolonged dual antiplatelet therapy?	JACC: Cardiovascular Interventions	Editorial
54	Kohno	2015	Report of the American Heart Association (AHA) Scientific Sessions 2014, Chicago	Circulation Journal	Commentary
55	Koppara	2015	Optical coherence tomography surveillance following drug-eluting stent implantation	Minerva Cardioangiologica	Review
56	Lavi	2015	Biodegredable stent platforms–Are we heading in the right direction?	Canadian Journal of Cardiology	Editorial
57	Lee	2015	Bleeding risks are in the eye of the beholder	ACP Journal Club	Commentary
58	Lee	2014	Dual Antiplatelet Therapy for Coronary Artery Disease	Circulation Journal	Review
59	Lemesle	2015	Dual antiplatelet therapy and non-cardiovascular mortality	The Lancet	Commentary
60	Lhermusier	2015	Prasugrel hydrochloride for the treatment of acute coronary syndromes	Expert opinion on pharmacotherapy	Review
61	Liou	2015	Optimal duration of dual antiplatelet therapy following drug-eluting stents implantation: A meta-analysis of 7 randomised controlled trials	International journal of cardiology	Article
62	Lipkin	2014	1 out of a hundred patient will benefit from extended dual Rx	NEJM	Commentary
63	Liu	2015	P2Y12 receptor inhibitors for secondary prevention of ischemic stroke	Expert opinion on pharmacotherapy	Review
64	Liu	2015	Percutaneous coronary intervention strategies and prognosis for graft lesions	Experimental and	Article

For peer review only

			following coronary artery bypass grafting	Therapeutic Medicine	
65	Madhavan	2015	Post-PCI Antithrombotic Therapy in Patients Requiring Long-Term Anticoagulation	Current cardiology reports	Review
66	Marrs	2015	Duration of Dual Antiplatelet Therapy after Drug-Eluting Stents	NEJM	Commentary
67	Matteau	2015	Balancing Long-Term Risks of Ischemic and Bleeding Complications after Percutaneous Coronary Intervention with Drug-Eluting Stents	The American journal of cardiology	Article
68	Matthews	2015	Persistence with secondary prevention medications after acute myocardial infarction: Insights from the TRANSLATE-ACS study	American Heart Journal	Article
69	Mauri	2015	Duration of Dual Antiplatelet Therapy after Drug-Eluting Stents -Author's reply	NEJM	Commentary
70	McKavanagh	2015	A Review of the Key Clinical Trials of 2014	Cardiology and therapy	Review
71	McMillan	2014	Nice slant	New England Journal of Medicine	Commentary
72	Mega	2015	Pharmacology of antithrombotic drugs: an assessment of oral antiplatelet and anticoagulant treatments	The Lancet	Review
73	Mehran	2015	DAPT Duration After DES: What Is the “Mandatory” Duration?	Journal of the American College of Cardiology	Editorial
74	Meneses	2014	About DAPT trial	New England Journal of Medicine	Commentary
75	Moschonas	2015	Protease-activated receptor-1 antagonists in long-term antiplatelet therapy. Current state of evidence and future perspectives	International journal of cardiology	Review

For peer review only

76	Mukherjee	2015	After drug-eluting stent placement, 6 months of dual antiplatelet therapy was noninferior to 12 months	Annals of Internal Medicine	Commentary
77	Navarese	2015	Optimal duration of dual antiplatelet therapy after percutaneous coronary intervention with drug eluting stents: meta-analysis of randomised controlled trials	BMJ	Article
78	Palmerini	2015	Mortality in patients treated with extended duration dual antiplatelet therapy after drug-eluting stent implantation: a pairwise and Bayesian network meta-analysis of randomised trials	The Lancet	Article
79	Papadimitriou	2015	Triple Antithrombotic Therapy: Is it Time to Drop the Aspirin?	Hospital Chronicles	Review
80	Parmar	2014	Error in Study Procedures!	NEJM	Commentary
81	Price	2015	The Optimal Duration of Dual Antiplatelet Therapy After Drug-Eluting Stent Implantation: Chasing a Mirage	Journal of the American College of Cardiology	Editorial
82	Raffoul	2015	Dual antiplatelet therapy duration after the placement of a drug-eluting stent: what are the data?	Current treatment options in cardiovascular medicine	Review
83	Rao	2015	The Conundrum of Reducing Ischemic and Bleeding Events After PCI∗	Journal of the American College of Cardiology	Editorial
84	Reejhsinghani	2015	Prevention of stent thrombosis: challenges and solutions	Vasc Health Risk Manag	Review
85	Rinfret	2015	Percutaneous Coronary Intervention: Finally Mature Enough	Journal of the American College of Cardiology	Editorial
86	Robbins	2015	Periprocedural management of aspirin during colonoscopy: a survey of practice patterns in the United States	Gastrointestinal endoscopy	Article

For peer review only

87	Rohla	2015	Double or triple antithrombotic combination therapy in patients who need anticoagulation and antiplatelet therapy in parallel	European Heart Journal-Cardiovascular Pharmacotherapy	Review
88	Ruparelia	2015	Dual antiplatelet therapy following drug-eluting stent implantation: how long is long enough?	Expert review of cardiovascular therapy	Editorial
89	Sabouret	2015	Dual antiplatelet therapy: optimal timing, management, and duration	European Heart Journal-Cardiovascular Pharmacotherapy	Review
90	Samardzic	2015	Temporal changes of platelet reactivity after coronary stenting–a thing to think about	American Journal of Cardiology	Commentary
91	Schiele	2015	mpact of prolonged dual antiplatelet therapy after acute myocardial infarction on 5-year mortality in the FAST-MI 2005 registry	International journal of cardiology	Article
92	Schulz-Schupke	2015	ISAR-SAFE: a randomized, double-blind, placebo-controlled trial of 6 vs. 12 months of clopidogrel therapy after drug-eluting stenting	European Heart Journal	Article
93	Secemsky	2015	Comparison of Short-and Long-Term Cardiac Mortality in Early Versus Late Stent Thrombosis (from Pooled PROTECT Trials)	The American journal of cardiology	Article
94	Shimohama	2015	Intrastent Thrombus - What You See Is What You Get?	Circulation Journal	Editorial
95	Simon	2015	Omeprazole, pantoprazole, and CYP2C19 effects on clopidogrel pharmacokinetic-pharmacodynamic relationships in stable coronary artery disease patients	European Journal of Clinical Pharmacology	Article
96	Sipahi	2015	Duration of Dual Antiplatelet Therapy after Drug-Eluting Stents	NEJM	Commentary
97	Sommer	2015	Stent Thrombosis: Current Management and Outcomes	Current treatment options in cardiovascular medicine	Review

For peer review only

98	Spencer	2015	Dual antiplatelets for 30 mo after drug-eluting stents reduced stent thrombosis and CV and cerebrovascular events.	ACP Journal Club	Commentary
99	Spencer	2015	Longer Versus Shorter Duration Dual-Antiplatelet Therapy After Drug-Eluting Stent PlacementA Systematic Review and Meta-analysisDuration of Dual-Antiplatelet Therapy After Drug-Eluting Stents	Annals of Internal Medicine	Article
100	Takeuchi	2015	Optimum duration of dual antiplatelet treatment could be decided using 64-MDCT: A new hint to treating patients with stents	IJC Heart & Vasculature	Others
101	Thomas	2015	The future of P2Y12 receptor antagonists	Platelets	Review
102	Tomoda	2015	Duration of Dual Antiplatelet Therapy after Drug-Eluting Stents	NEJM	Commentary
103	Toyota	2015	Meta-analysis of Long-term Clinical Outcomes of Everolimus-eluting Stents	The American journal of cardiology	Article
104	Tremmel	2015	Late breaking trials of 2014 in coronary artery disease: Commentary covering ACC, EuroPCR, SCAI, TCT, ESC, and AHA	Catheterization and Cardiovascular Interventions	Commentary
105	Tsoumani	2015	Evaluating the bioequivalence of clopidogrel generic formulations	Current medical research and opinion	Editorial
106	Valgimigli	2015	Duration of dual antiplatelet therapy after drug-eluting stent implantation: will we ever reach a consensus?	European Heart Journal	Editorial
107	Van de Werf	2015	The year in cardiology 2014: acute coronary syndromes	European Heart Journal	Review
108	Vetrovec	2015	Another Challenge for the Presumed Safety Advantage of Bare Metal Stents	Catheterization and Cardiovascular Interventions	Editorial
109	Vranckx	2015	Peri-procedural use of rivaroxaban in elective percutaneous coronary intervention to treat stable coronary artery disease. The XPLORER trial	Thrombosis and Haemostasis	Article
110	Waksman	2015	Do you still have an appetite for a short DAPT trial?	Cardiovascular Revascularization Medicine	Editorial

For peer review only

111	Watanabe	2015	Antiplatelet therapy discontinuation and the risk of serious cardiovascular events after coronary stenting: observations from the CREDO-Kyoto Registry Cohort-2	PLoS ONE	Article
112	Wiviott	2015	Clinical evidence for oral antiplatelet therapy in acute coronary syndromes	The Lancet	Review
113	Yamaji	2015	Long-term Outcomes after Coronary Stent Implantation in Patients Presenting with versus without Acute Myocardial Infarction (An observation from Coronary Revascularization Demonstrating Outcome Study-Kyoto Registry Cohort-2)	The American journal of cardiology	Article
114	Yang	2015	Current antiplatelet agents: place in therapy and role of genetic testing	Journal of thrombosis and thrombolysis	Review
115	Yeh	2015	Benefits and risks of extended duration dual antiplatelet therapy after PCI in patients with and without acute myocardial infarction	Journal of the American College of Cardiology	Article
116	Yeh	2015	Dual Antiplatelet Platelet Therapy Duration Following Coronary Stenting	Journal of the American College of Cardiology	Editorial
117	Yeh	2015	Dual antiplatelet therapy duration and mortality–Authors' reply	The Lancet	Commentary
118	Yeh	2015	Close encounters with errors of the second kind: evaluating risks and benefits of long-term dual antiplatelet therapy	European Heart Journal	Editorial

For peer review only

Appendix 2: Content of the scholarly and public attention surrounding the DAPT study by source (n = 425)

Category	Overall n=425	Scientific communication 118 (27.7)	News 12 (2.8)	Blogs 3 (0.7)	Facebook posts 189 (44.4)	Tweets 75 (17.6)	YouTube 8 (1.9)	DAPT Website 20 (4.7)
Text favourable about the prolonged treatment	81 (19.1)	28 (23.7)	9 (75.0)	-	9 (4.8)	11 (14.7)	(100)	16 (80.0)
Text uncertain, with inappropriate mention of mortality	19 (4.5)	13 (11.0)	3 (25.0)	2 (66.7)	-	-	-	1 (5.0)
Electronic link	151 (35.5)	-	-	-	113 (59.8)	38 (50.6)	-	-
Referenced with no message	13 (3.1)	1 (0.8)	-	-	10 (5.3)	2 (2.7)	-	-
Text uncertain, with no mention of mortality	100 (23.5)	37 (31.4)	-	1 (33.3)	48 (25.4)	13 (17.3)	-	1 (5.0)
Text uncertain, with appropriate mention of mortality	34 (8.0)	29 (24.6)	-	-	1 (0.5)	3 (4.0)	-	1 (5.0)
Text not favourable about the prolonged treatment	15 (3.5)	3 (2.5)	-	-	8 (4.2)	3 (4.0)	-	1 (5.0)
Text not favourable about the prolonged treatment and critical of the authors' interpretation	12 (3.0)	7 (6.0)	-	-	-	5 (6.7)	-	-

For peer review only

STROBE 2007 (v4) Statement—Checklist of items that should be included in reports of *cross-sectional studies*

Section/Topic	Item #	Recommendation	Reported on page #
**Title and abstract**	1	(*a*) Indicate the study's design with a commonly used term in the title or the abstract	1
		(*b*) Provide in the abstract an informative and balanced summary of what was done and what was found	2
**Introduction**			**4**
Background/rationale	2	Explain the scientific background and rationale for the investigation being reported	4, 5
Objectives	3	State specific objectives, including any prespecified hypotheses	5
**Methods**			**6**
Study design	4	Present key elements of study design early in the paper	6
Setting	5	Describe the setting, locations, and relevant dates, including periods of recruitment, exposure, follow-up, and data collection	6
Participants	6	(*a*) Give the eligibility criteria, and the sources and methods of selection of participants ***(no participants were involved in this study. Unit of study was the items disseminating DAPT study).***	7
Variables	7	Clearly define all outcomes, exposures, predictors, potential confounders, and effect modifiers. Give diagnostic criteria, if applicable	7
Data sources/ measurement	8*	For each variable of interest, give sources of data and details of methods of assessment (measurement). Describe comparability of assessment methods if there is more than one group	7, 8
Bias	9	Describe any efforts to address potential sources of bias	*No*
Study size	10	Explain how the study size was arrived at	6
Quantitative variables	11	Explain how quantitative variables were handled in the analyses. If applicable, describe which groupings were chosen and why	NA
Statistical methods	12	(*a*) Describe all statistical methods, including those used to control for confounding	8
		(*b*) Describe any methods used to examine subgroups and interactions	
		(*c*) Explain how missing data were addressed	
		(*d*) If applicable, describe analytical methods taking account of sampling strategy	
		(*e*) Describe any sensitivity analyses	
**Results**			9

For peer review only

Participants	13*	(a) Report numbers of individuals at each stage of study—eg numbers potentially eligible, examined for eligibility, confirmed eligible, included in the study, completing follow-up, and analysed (in flow diagram)	9, 17
		(b) Give reasons for non-participation at each stage (in flow diagram)	17
		(c) Consider use of a flow diagram	9
Descriptive data	14*	(a) Give characteristics of study participants (eg demographic, clinical, social) and information on exposures and potential confounders (Unit of study was the items disseminating DAPT study)	9
		(b) Indicate number of participants with missing data for each variable of interest	
Outcome data	15*	Report numbers of outcome events or summary measures	9, 10
Main results	16	(*a*) Give unadjusted estimates and, if applicable, confounder-adjusted estimates and their precision (eg, 95% confidence interval). Make clear which confounders were adjusted for and why they were included	9, 10
		(*b*) Report category boundaries when continuous variables were categorized	
		(*c*) If relevant, consider translating estimates of relative risk into absolute risk for a meaningful time period	
Other analyses	17	Report other analyses done—eg analyses of subgroups and interactions, and sensitivity analyses	NA
**Discussion**			11
Key results	18	Summarise key results with reference to study objectives	11
Limitations	19	Discuss limitations of the study, taking into account sources of potential bias or imprecision. Discuss both direction and magnitude of any potential bias	11, 12
Interpretation	20	Give a cautious overall interpretation of results considering objectives, limitations, multiplicity of analyses, results from similar studies, and other relevant evidence	11, 12
Generalisability	21	Discuss the generalisability (external validity) of the study results	11
**Other information**			12-14
Funding	22	Give the source of funding and the role of the funders for the present study and, if applicable, for the original study on which the present article is based	13

*Give information separately for cases and controls in case-control studies and, if applicable, for exposed and unexposed groups in cohort and cross-sectional studies.

Note: An Explanation and Elaboration article discusses each checklist item and gives methodological background and published examples of transparent reporting. The STROBE checklist is best used in conjunction with this article (freely available on the Web sites of PLoS Medicine at http://www.plosmedicine.org/, Annals of Internal Medicine at http://www.annals.org/, and Epidemiology at http://www.epidem.com/). Information on the STROBE Initiative is available at www.strobe-statement.org.

For peer review only

BMJ Open

Dissemination of 2014 Dual Anti-platelet Therapy (DAPT) trial results: A systematic review of scholarly and media attention over 7 months

Journal:	*BMJ Open*
Manuscript ID	bmjopen-2016-014503.R2
Article Type:	Research
Date Submitted by the Author:	10-Aug-2017
Complete List of Authors:	Sharp, Melissa K; Columbia University, Mailman School of Public Health; INSERM UMR 1153, Center of Research in Epidemiology and Statistics Sorbonne Paris Cité, Center of Epidemiology and Clinical Research<br>HANEEF, Romana; INSERM UMR 1153, Center of Research in Epidemiology and Statistics Sorbonne Paris Cité, Center of Epidemiology and Clinical Research; University of Paris Descartes<br>Ravaud, Philippe; INSERM UMR 1153, Center of Research in Epidemiology and Statistics Sorbonne Paris Cité, Center of Epidemiology and Clinical Research; University of Paris Descartes<br>Boutron, Isabelle; INSERM UMR 1153, Center of Research in Epidemiology and Statistics Sorbonne Paris Cité, Center of Epidemiology and Clinical Research; University of Paris Descartes
**Primary Subject Heading**:	Epidemiology
Secondary Subject Heading:	Qualitative research, Public health, Research methods, Communication
Keywords:	DAPT Therapy, Misleading interpretation, Mortality, Public attention, Critical opinion

SCHOLARONE™
Manuscripts

Dissemination of 2014 Dual Anti-platelet Therapy (DAPT) trial results: A systematic review of scholarly and media attention over 7 months

Melissa K Sharp• (1,2), Romana Haneef• (2,3,4), Philippe Ravaud (1,2,3,4,5), Isabelle Boutron*(2,3,4,5)

(1) Columbia University, Mailman School of Public Health, New York, NY, USA

(2) METHODS Team, Center of Research in Epidemiology and Statistics Sorbonne Paris Cité, UMR 1153, INSERM, Paris, France.

(3) University Paris Descartes, Sorbonne Paris Cité, Paris, France

(4) Centre d'Épidémiologie Clinique, AP-HP (Assistance Publique des Hôpitaux de Paris), Hôpital Hôtel Dieu, Paris, France

(5) Cochrane France, Paris, France

• These authors contributed equally to this work as a first author.

*Corresponding author:

Isabelle Boutron

Centre d'Epidémiologie Clinique

Hôpital Hôtel Dieu,

1, Place du parvis Notre Dame

75004 Paris Cedex 4

E-mail : isabelle.boutron@aphp.fr

Tel : 00 33 1 42 34 78 33

Fax : 00 33 1 42 34 87 90

For peer review only - http://bmjopen.bmj.com/site/about/guidelines.xhtml

Abstract

Objective: To explore how the results from the 2014 DAPT trial were disseminated to the scientific community and online media.

Design: A cross-sectional study of scholarly and public attention surrounding the DAPT study.

Settings: Data were collected from the ISI Web of Knowledge, Google Scholar, PubMed Commons, EurekAlert, the DAPT study website (www.daptstudy.org), and the *New England Journal of Medicine* website *(for scholarly attention)* and Altmetric Explorer, Snap Bird, YouTube *(for public attention)* citing DAPT study results appearing from November 16, 2014 to June 10, 2015.

Participants: No participants were involved in this study.

Main outcome measure: Proportion of contents highlighting the increased risk of mortality and critical to the author's interpretation of the results.

Results: We identified 425 items reported by 7 sources; 164 (39%) disseminated the authors' interpretation via an electronic link or a reference, with no additional text. Among 81 items (19%), the message favoured prolonged treatment and consequently overstated the article conclusions. Among 119 items (28%), the text was uncertain about the benefit of prolonged treatment but was reported with no or inappropriate mention of increased risk of mortality. Only 34 items (8%) were uncertain about the benefit of prolonged treatment and mentioned increased risk of mortality. In all, 27 items (6%) did not favour prolonged treatment, and only 12 of these (3%) clearly raised some concerns about the reporting of increased risk of death.

Conclusion: Dissemination of the DAPT study results to the scientific community and on different media sources rarely criticized the interpretation of the study results.

For peer review only

For peer review only - http://bmjopen.bmj.com/site/about/guidelines.xhtml

Strengths and limitation of this study

- Our method involved a broad search strategy, ensured to capture an extensive and representative sample of contents citing the 2014 DAPT trial for both scholarly and public attention.
- Our systematic approach to analyze the text of contents provides a comprehensive overview of dissemination of the study results.
- This study focused on only a specific trial publication and results are not generalizable to other studies.

For peer review only

For peer review only - http://bmjopen.bmj.com/site/about/guidelines.xhtml

INTRODUCTION

The development of optimal coronary stent replacement has progressed rapidly over recent years [1]. In the United States, almost 700,000 stents are placed every year and there is an increasing trend for its use in Europe [2]. Dual antiplatelet therapy (DAPT) (i.e., P2Y12-receptor inhibitor combined with aspirin) is recommended after placement of coronary stents to prevent thrombotic complications [3]. The optimal duration of DAPT has been debated [4-8].

In December 2014, the Harvard Clinical Research Institute (HCRI) released the results of the DAPT study, the largest international randomized controlled trial to date [9]. The trial aimed to determine the benefits and risks of continuing DAPT beyond 1 year after placement of a coronary stent [9]. A total of 9,961 adult patients were randomly assigned to continue thienopyridine treatment or to receive a placebo for 30 months. Continued therapy reduced the rate of stent thrombosis (0.4% vs.1.4%; p<0.001) and major adverse cardiovascular and cerebrovascular events (MACCEs) (2.1% vs. 4.1%; p<0.001), with an expected increase in the rate of moderate or severe bleeding (2.5% vs. 1.6%; p=0.001) [9]. However, continued therapy was also associated with an increase of 36% in all-cause mortality (2.0% vs. 1.5%; hazard ratio 1.36 [95% CI 1.00 to 1.85]; P=0.05).

The results of the DAPT study were published in the *New England Journal of Medicine* (*NEJM*) [9] after their presentation at the American Health Association Conference, in November 2014. However, the reporting of the results raised some concerns [10, 11]. Particularly, the abstract conclusions did not mention the increased risk of mortality. Furthermore, the discussion included explanations based on post-hoc analyses to clear the role of prolonged thienopyridine treatment in this increased risk of mortality. For this purpose, the authors had split the analysis by cause of death, which was not powered to show a statistically significant difference. They focused on the increase in cancer-related death (0.62% vs 0.28%,

For peer review only - http://bmjopen.bmj.com/site/about/guidelines.xhtml

p = 0.02). The results were interpreted as being related to an imbalance at baseline in patients with a history of cancer before enrolment (9.8% vs 9.5%). To confirm, the authors performed a post-hoc analysis excluding all deaths that could be related to cancer diagnosed before enrolment. Consequently, the results became statistically non-significant (0.50% vs 0.28%, p=0.11). This post-hoc exclusion of patients with an event is questionable.

We aimed to explore how the authors' interpretation of results from the DAPT trial was disseminated to the scientific community and online media and to assess whether this interpretation was criticized or not.

For peer review only

For peer review only - http://bmjopen.bmj.com/site/about/guidelines.xhtml

METHODS

We performed a cross-sectional study of scholarly and public attention surrounding the DAPT study.

Identification of scholarly and public attention surrounding the DAPT study

Scholarly attention

On June 2015, we searched the following electronic databases to identify responses to the DAPT study: ISI Web of Knowledge, Google Scholar, and PubMed Commons. We also searched the comments and citing articles on the *NEJM* website for the original article [9].

Public attention

We searched Altmetric Explorer [12-15] to identify all online attention (news, blogs, Twitter, Facebook, Google+, Mendeley, CiteULike) given to the DAPT study. Each identified social media source was then systematically evaluated to determine whether other posts were not captured by Altmetric Explorer. In addition, each original tweet was reviewed to find retweets, replies and favourites. Since Altmetric.com captures only tweets attached to the DOI (Digital Object Identifier) of the original DAPT article, we also used snapbird.org, a search engine that can search an individual Twitter account by using the *NEJM*'s Twitter account and the search terms "DAPT" and "dual antiplatelet therapy". We also searched EurekAlert! (a free online database for science press releases, www.eurekalert.org) for press releases dedicated to the DAPT study; YouTube (search terms "DAPT" and "dual antiplatelet therapy"); and pages dedicated to patients, clinicians and media at the DAPT study website (http://www.daptstudy.org).

For peer review only - http://bmjopen.bmj.com/site/about/guidelines.xhtml

Eligibility criteria

Two researchers (MS, RH) screened all items retrieved and selected all English-language items that cited the DAPT study and were released from November 16, 2014 to June 10, 2015. Any disagreements were resolved by discussion to reach consensus.

Content of scholarly and public attention surrounding the DAPT study

Two researchers (MS, RH) read the items from each source independently and evaluated them by using a preliminarily tested extraction form. Disagreements were resolved by discussion to reach consensus. If needed, a third researcher (IB) appraised the content.

We determined whether the source consisted of a reference or a link to the *NEJM* article reporting the DAPT study only or was a text commenting on the DAPT study. For a text commenting on the DAPT study, we checked whether the original study authors were involved in writing the text or not. Our main outcome of interest was the proportion of contents highlighting the increased risk of mortality and critical to the author's interpretation of the results. We determined whether

- the primary efficacy outcomes (i.e., stent thrombosis and MACCE) were reported
- the safety outcomes related to moderate or severe bleeding were reported
- the increased risk of mortality with prolonged treatment was reported
- the authors' explanation clearing the responsibility of prolonged treatment in the increased risk of mortality was reported or criticized
- the content of the text was 1) favouring the prolonged treatment and consequently overstating the article conclusion, 2) uncertain about the benefit of the prolonged treatment (i.e., statement of both the beneficial effect, and increased risk of bleeding, text ending with a question mark, use of "may or might" or reporting that the study needs further research), or 3) not favouring the prolonged treatment [16].

For peer review only - http://bmjopen.bmj.com/site/about/guidelines.xhtml

Overall, we classified the sources based on the text of contents as follows:

1. Text favouring the prolonged treatment
2. Text uncertain (about the benefit of prolonged treatment) with inappropriate mention of mortality
3. Text neutral/uncertain (about the benefit of prolonged treatment) with no mention of mortality
4. Electronic link or referenced with no message
5. Text uncertain (about the benefit of prolonged treatment) with appropriate mention of mortality
6. Text not favouring the prolonged treatment
7. Text not favouring the prolonged treatment and critical of the authors' interpretation

Statistical analysis

We calculated frequencies and percentages (%) for qualitative variables and median (interquartile range) for quantitative variables.

For peer review only - http://bmjopen.bmj.com/site/about/guidelines.xhtml

RESULTS

Identification of scholarly and public attention surrounding the DAPT study

From all sources, we selected and appraised 425 items: 118 scientific communications, 12 news items, 3 blogs, 189 Facebook posts or comments, 75 tweets or replies, 8 videos on YouTube, 14 DAPT media pages, 5 DAPT website pages and 1 video on the DAPT website (Figure 1). The original study authors were directly involved in 35 items. Details of 118 scientific communications are in *Appendix 1*.

Reporting of the content

The texts of contents are described in Figure 2 (overall) and Figure 3 (by source). Overall, 164 items (39%) involved disseminating the authors' reporting and interpretation via an electronic link (n=151, 36%) or reference (n=13; 3%), with no additional text or message. Among 81 items (19%), the message favoured the prolonged treatment and therefore overstated the article conclusions. For example, the DAPT study website dedicated to patients reported that "*It is important that patients who currently take a thienopyridine anti-clotting medication (clopidogrel or prasugrel) do not stop taking their medication. […] The benefits of continuing dual antiplatelet therapy for one year, according to current guidelines, far outweigh the risks*." Among 153 items (36%), the text was uncertain about the benefit of prolonged treatment but was reported with no mention of the increased risk of mortality (n=100, 24%) or the authors' explanation clearing the responsibility of prolonged treatment (n=19; 4.5%). Overall, 34 items (8%) were uncertain about the benefit of prolonged treatment but mentioned the increased risk of mortality. Only 27 (6%) did not favour prolonged treatment and only 12 of these (3%) clearly raised some concerns about the reporting of the increased risk of death. Further information on items by source is in *Appendix 2*.

For peer review only - http://bmjopen.bmj.com/site/about/guidelines.xhtml

Overall, 136 items (32%) reported efficacy outcomes (i.e., stent thrombosis and MACCEs), 127 (30%) safety outcomes and 113 (27%) both efficacy and safety outcomes.

A total of 100 items (24%) did not mention mortality, but when mortality was mentioned, in 19 items (5%), it was reported with the authors' justification for prolonged treatment.

For peer review only

For peer review only - http://bmjopen.bmj.com/site/about/guidelines.xhtml

DISCUSSION

We describe the dissemination of the 2014 DAPT study findings in scientific community and to the public via different sources such as news, blogs, and social media. Our assessment of 425 items disseminating the DAPT study results showed that only 8% of the items mentioned some uncertainty about the benefit of prolonged treatment and included a mention of the increased risk of mortality. Furthermore, only 12 items (3%) clearly raised some concerns about the reporting of the increased risk of death. This study adds to the burgeoning literature on the biased dissemination of research results. Previous studies have focused on publication bias [17], selective reporting of outcomes [17-22], and spin [19, 23, 24].

However, this is the first study to our knowledge to focus on both scholarly and public dissemination of study results. Our study highlighted an unmet need of scientific communication in the media, whose importance in dissemination of scientific data is becoming increasingly relevant. These findings could be helpful for the entire community for better understanding how scientific knowledge is disseminated.

Our approach involved a broad search strategy and multiple search engines, which ensured the capture of an extensive and representative sample of contents discussing the DAPT study results. Each social media item from Altmetric was systematically reviewed for additional content that may have been missed, and several different search engines were used. We captured items that were published over the course of many months, which highlighted the perpetuation and continuation of the dissemination of the authors' interpretations. The inclusion period for sources seemed to be more than sufficient because tweets linked to scientific articles have been shown to taper off well before our cut-off point (7 months) [25]. In addition, 2 independent researchers assessed each source by using a standardized data extraction form and disagreements were resolved by consensus.

For peer review only - http://bmjopen.bmj.com/site/about/guidelines.xhtml

However, our study has some limitations. First, this study focused on only a specific trial publication and results are not generalizable to other studies. However, the article we focused on was among the top 5 of all research outputs and within the 99th percentile of articles on Altmetric. Second, the data extraction involved some subjectivity; however, we tried to address this by using a standardized data extraction form and independent assessment as well as consensus among 2 researchers. Third, despite our best efforts, we cannot ensure that our search strategy was all-encompassing because of the breadth of social media. Finally, we did not explore the balance between efficacy and safety outcomes with DAPT treatment.

Our aim was not to resolve the controversy about DAPT duration and this debate is still ongoing. The OPITUDAL trial did not find an increased risk of death with the prolonged treatment; on the contrary, the risk of death was lower with the prolonged treatment [26]. Several meta-analyses found conflicting results [4, 5, 8, 27, 28]. The researchers involved in the DAPT trial concluded in a meta-analysis published in *The Lancet* that prolonged DAPT duration was not associated with a difference in risk of all-cause mortality [29]. Three meta-analyses, published later by different teams, showed prolonged DAPT associated with increased risk of all-cause mortality [4, 5, 8]. More recently, other meta-analyses did not find a statistically significant increase in all-cause mortality [27, 28]. Most of these meta-analyses warranted further research with extended DAPT.

However, these results are difficult to interpret because of different definitions of short (1, 3, 6, or 12 months) and extended (6, 12, 24 or > 24 months) durations, which varied across studies. Furthermore, different durations of follow-up and types of stents could also influence the results.

CONCLUSIONS

For peer review only - http://bmjopen.bmj.com/site/about/guidelines.xhtml

Dissemination of the DAPT study results to the scientific community and on different media sources rarely criticized the interpretation of the study results.

Supplementary Data

Appendix 1: Detail of 118 scientific communications

Appendix 2: Content of scholarly and public attention surrounding the DAPT study by source.

For peer review only

For peer review only - http://bmjopen.bmj.com/site/about/guidelines.xhtml

Acknowledgements

We thank Elise Diard for help in creating Figure 2. We acknowledge support from Altmetric for free access to "Altmetric Explorer". We acknowledge the assistance in English language proofreading by Laura Smales (BioMedEditing, Toronto, Canada).

Isabelle Boutron and Philippe Ravaud submitted a letter to the *NEJM* following the publication of the DAPT study to highlight the inadequate reporting in the abstract conclusions, but the letter was rejected.

Contributors

Study conception, design, selection of contents and data extraction: MS, RH. Study conception and design: MS, RH, IB. Selection of contents, data extraction: MS, RH. Analysis of data and interpretation of results: RH, PR, IB. Contributed to the writing of the manuscript: MS, RH, PR, IB. All authors read and approved the final manuscript.

Funding Source

This study did not receive any funding.

Competing interests

None declared.

Ethical approval

Not needed

For peer review only - http://bmjopen.bmj.com/site/about/guidelines.xhtml

Data sharing

All relevant data are included in this manuscript. Details of text content are available upon request for academic researchers.

Open Access

This is an Open Access article distributed in accordance with the Creative Commons Attribution Non Commercial (CC BY-NC 4.0) license, which permits others to distribute, remix, adapt, build upon this work non-commercially, and license their derivative works on different terms, provided the original work is properly cited and the use is non-commercial. See: http://creativecommons.org/licenses/by-nc/4.0/.

For peer review only

For peer review only - http://bmjopen.bmj.com/site/about/guidelines.xhtml

References

1. Wilson WM. Advances in coronary stent technology: current expectations and new developments. *Research Reports in Clinical Cardiology* 2013.
2. Mozaffarian D, Benjamin EJ, Go AS, et al. Heart disease and stroke statistics--2015 update: a report from the American Heart Association. *Circulation* 2015; **131**: e29-322.
3. Palmerini T, Dangas G, Mehran R, et al. Predictors and Implications of Stent Thrombosis in Non–ST-Segment Elevation Acute Coronary Syndromes: The ACUITY Trial. *Circulation: Cardiovascular Interventions* 2011; **4**: 577-84.
4. Palmerini T, Benedetto U, Bacchi-Reggiani L, et al. Mortality in patients treated with extended duration dual antiplatelet therapy after drug-eluting stent implantation: a pairwise and Bayesian network meta-analysis of randomised trials. *The Lancet* 2015.
5. Spencer FA, Prasad M, Vandvik PO, Chetan D, Zhou Q, Guyatt G. Longer- Versus Shorter-Duration Dual-Antiplatelet Therapy After Drug-Eluting Stent PlacementA Systematic Review and Meta-analysisDuration of Dual-Antiplatelet Therapy After Drug-Eluting Stents. *Annals of Internal Medicine* 2015; **163**: 118-26.
6. Levine GN, Bates ER, Blankenship JC, et al. 2011 ACCF/AHA/SCAI Guideline for Percutaneous Coronary InterventionA Report of the American College of Cardiology Foundation/American Heart Association Task Force on Practice Guidelines and the Society for Cardiovascular Angiography and Interventions. *Journal of the American College of Cardiology* 2011; **58**: e44-e122.
7. Windecker S, Kolh P, Alfonso F, et al. 2014 ESC/EACTS Guidelines on myocardial revascularization. *The Task Force on Myocardial Revascularization of the European Society of Cardiology (ESC) and the European Association for Cardio-Thoracic Surgery (EACTS)Developed with the special contribution of the European Association of Percutaneous Cardiovascular …* 2014; **35**: 2541-619.
8. Navarese EP, Andreotti F, Schulze V, et al. Optimal duration of dual antiplatelet therapy after percutaneous coronary intervention with drug eluting stents: meta-analysis of randomised controlled trials. *British Medical Journal* 2015; **350**.
9. Mauri L, Kereiakes DJ, Yeh RW, et al. Twelve or 30 Months of Dual Antiplatelet Therapy after Drug-Eluting Stents. *New England Journal of Medicine* 2014; **371**: 2155-66.
10. Lipkin D. 1 out of a hundred patient will benefit from extended dual Rx. 2014.
11. Gupta R. Dual antiplatelets :Walking on a tight rope. 2014.
12. Altmetric, https://www.altmetric.com/whatwedo.php. 2012.
13. Haustein S, Costas R, Larivière V. Characterizing Social Media Metrics of Scholarly Papers: The Effect of Document Properties and Collaboration Patterns. *PLoS ONE* 2015; **10**: e0120495.
14. Liu CL, Xu YQ, Wu H, Chen SS, Guo JJ. Correlation and Interaction Visualization of Altmetric Indicators Extracted From Scholarly Social Network Activities: Dimensions and Structure. *Journal of Medical Internet Research* 2013; **15**: e259.
15. Thelwall M, Haustein S, Larivière V, Sugimoto CR. Do Altmetrics Work? Twitter and Ten Other Social Web Services. *PLoS ONE* 2013; **8**: e64841.
16. Yank V, Rennie D, Bero LA. Financial ties and concordance between results and conclusions in meta-analyses: retrospective cohort study. *BMJ* 2007; **335**: 1202-5.
17. Dwan K, Altman DG, Arnaiz JA, et al. Systematic Review of the Empirical Evidence of Study Publication Bias and Outcome Reporting Bias. *PLoS ONE* 2008; **3**: e3081.
18. Riveros C, Dechartres A, Perrodeau E, Haneef R, Boutron I, Ravaud P. Timing and Completeness of Trial Results Posted at ClinicalTrials.gov and Published in Journals. *PLoS Med* 2013; **10**: e1001566.
19. Boutron I, Dutton S, Ravaud P, Altman DG. Reporting and interpretation of randomized controlled trials with statistically nonsignificant results for primary outcomes. *JAMA* 2010; **303**.

For peer review only - http://bmjopen.bmj.com/site/about/guidelines.xhtml

For peer review only

20. Tang E, Ravaud P, Riveros C, Perrodeau E, Dechartres A. Comparison of serious adverse events posted at ClinicalTrials.gov and published in corresponding journal articles. *BMC Medicine* 2015; **13**: 1-8.
21. Mathieu S, Boutron I, Moher D, Altman DG, Ravaud P. Comparison of registered and published primary outcomes in randomized controlled trials. *JAMA* 2009; **302**.
22. Chan A, Hróbjartsson A, Haahr MT, Gøtzsche PC, Altman DG. Empirical evidence for selective reporting of outcomes in randomized trials: Comparison of protocols to published articles. *JAMA* 2004; **291**: 2457-65.
23. Lazarus C, Haneef R, Ravaud P, Boutron I. Classification and prevalence of spin in abstracts of non-randomized studies evaluating an intervention. *BMC Medical Research Methodology* 2015; **15**: 1-8.
24. Yavchitz A, Boutron I, Bafeta A, et al. Misrepresentation of Randomized Controlled Trials in Press Releases and News Coverage: A Cohort Study. *PLoS Med* 2012; **9**: e1001308.
25. Eysenbach G. Can Tweets Predict Citations? Metrics of Social Impact Based on Twitter and Correlation with Traditional Metrics of Scientific Impact. *J Med Internet Res* 2011; **13**: e123.
26. Helft G, Steg PG, Le Feuvre C, et al. Stopping or continuing clopidogrel 12 months after drug-eluting stent placement: the OPTIDUAL randomized trial. *European Heart Journal* 2016; **37**: 365-74.
27. Basaraba JE, Barry AR. Short- versus standard-term dual antiplatelet therapy after percutaneous coronary intervention with drug-eluting stent implantation: A meta-analysis. *J Cardiol* 2017; **69**: 353-8.
28. D'Ascenzo F, Moretti C, Bianco M, et al. Meta-Analysis of the Duration of Dual Antiplatelet Therapy in Patients Treated With Second-Generation Drug-Eluting Stents. *The American journal of cardiology* 2016; **117**: 1714-23.
29. Elmariah S, Mauri L, Doros G, et al. Extended duration dual antiplatelet therapy and mortality: a systematic review and meta-analysis. *The Lancet* 2015; **385**: 792-8.

For peer review only

Figure 1: Flow diagram of identified scholarly and public attention surrounding the DAPT study

For peer review only

For peer review only - http://bmjopen.bmj.com/site/about/guidelines.xhtml

Figure 2: Content of scholarly and public attention surrounding the DAPT study (n = 425)

For peer review only

*Increased risk of mortality reported with the authors' questionable explanation clearing the responsibility of prolonged treatment in the increased risk

** Increased risk of mortality reported without any explanation

For peer review only - http://bmjopen.bmj.com/site/about/guidelines.xhtml

Figure 3: Content of scholarly and public attention surrounding the DAPT study by source (n = 425)

For peer review only

20

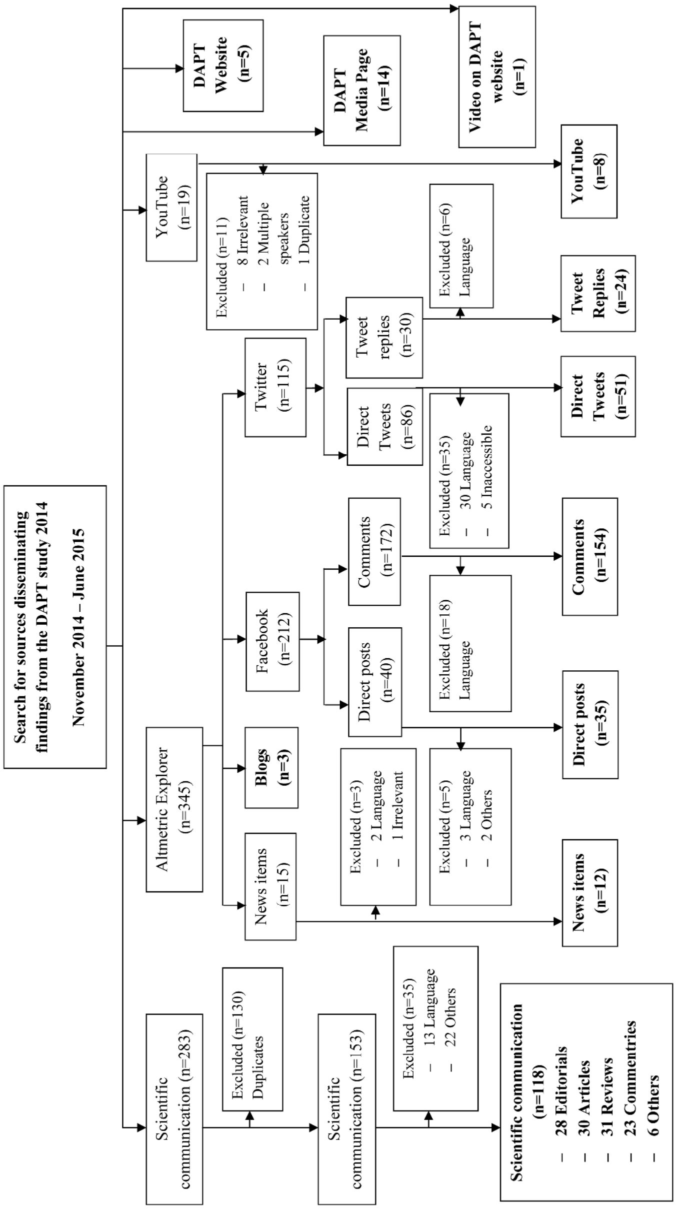

Flow diagram of identified scholarly and public attention surrounding the DAPT study

282x505mm (300 x 300 DPI)

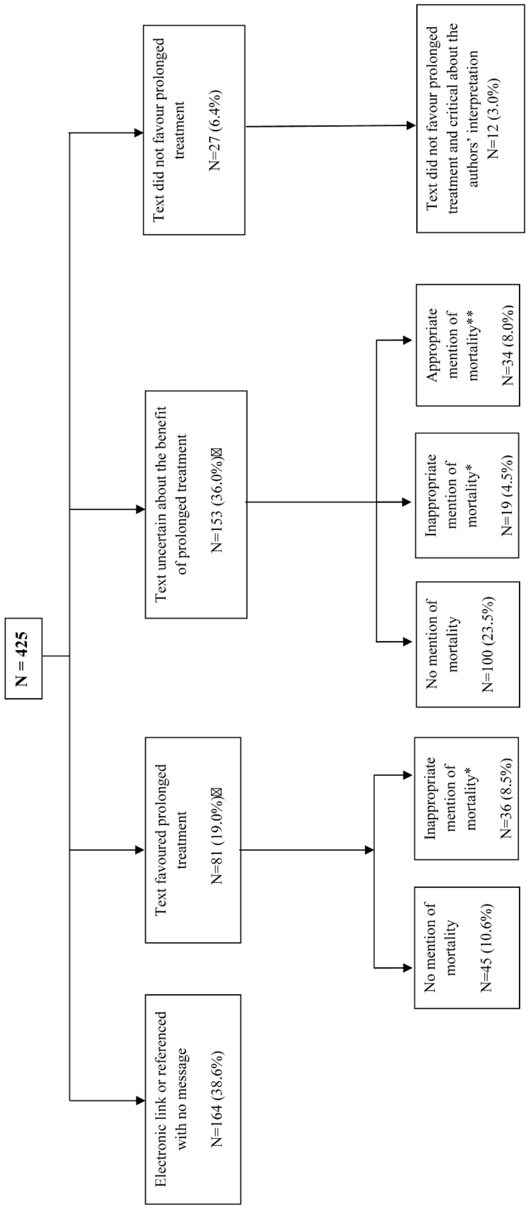

Content of the scholarly and public attention surrounding the DAPT study (n = 425)

119x276mm (300 x 300 DPI)

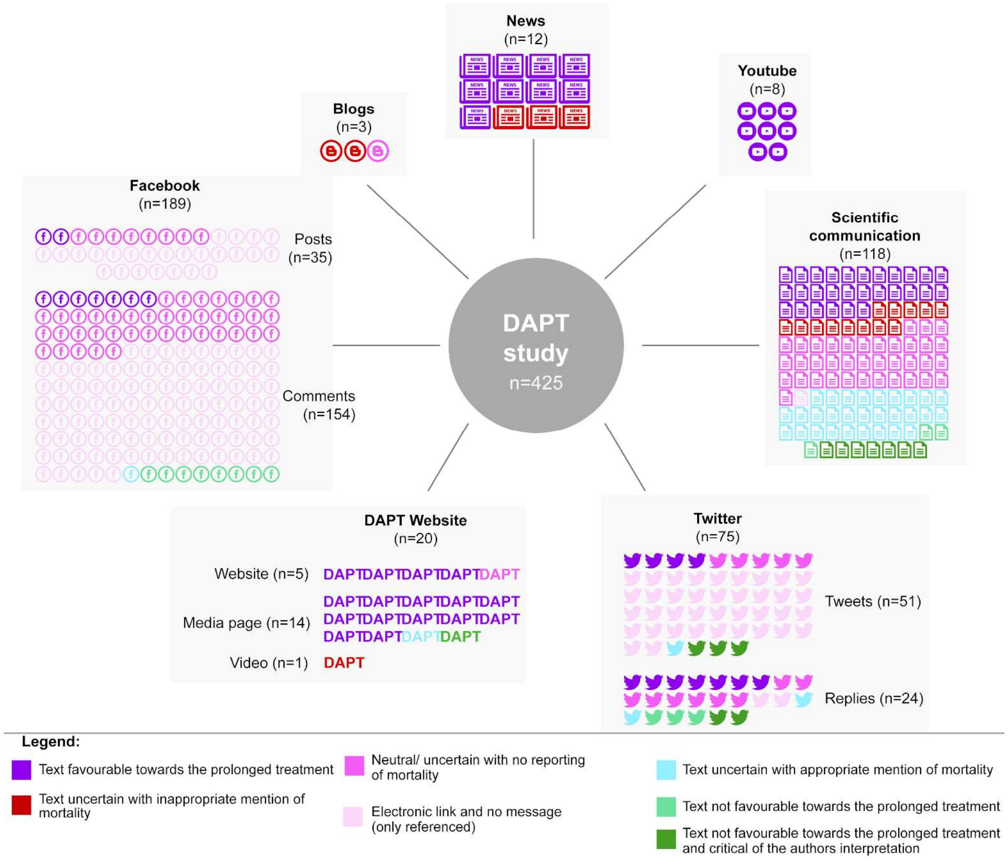

Content of the scholarly and public attention surrounding the DAPT study by source (n = 425)

256x219mm (300 x 300 DPI)

Appendix 1: Detail of 118 scientific communications					
S/No	First Author	Year	Title	Journal	Type of scientific contribution
1	Abo-salem	2015	Optimal duration of dual antiplatelet therapy after drug eluting stents: Meta-analysis of randomized trials	Cardiovascular Therapeutics	Article
2	Alfredsson	2015	Balancing the risks and benefits of long-term antiplatelet therapies for cardiovascular disease: clinical, research, and regulatory implications	J Am Heart Association	Editorial
3	AlJaroudi	2014	Review of Cardiovascular Literature	Journal of nuclear cardiology	Review
4	Angoulvant	2015	Dual antiplatelet therapy after acute coronary syndrome: a cardiologist-based optimal decision	Heart	Editorial
5	Aradi	2015	ATLANTIC: another reason to investigate the disconnect between stent thrombosis and mortality?	Thromosis & Haemostatis	Editorial
6	Auer	2015	Dual antiplatelet therapy duration and mortality	Lancet	Commentary
7	Becker	2015	Are at Least 12 Months of Dual Antiplatelet Therapy Needed for All Patients With Drug-Eluting Stents? Not All Patients With Drug-Eluting Stents Need at Least 12 Months of Dual Antiplatelet Therapy	Circulation	Editorial
8	Binder	2015	Duration of dual antiplatelet therapy after coronary artery stenting: where is the sweet spot between ischaemia and bleeding?	Europeaon Heart Journal	Editorial
9	Biondi-Zoccai	2015	Noncompliance and Cessation of Dual Antiplatelet Therapy After Coronary Stenting Looking at the Speck Rather Than Noticing the Log?	JACC-Cardiovascular Interventions	Editorial
10	Bonaca	2015	Long-term use of ticagrelor in patients with prior myocardial infarction	NEJM	Article
11	Brener	2015	Are at Least 12 Months of Dual Antiplatelet Therapy Needed for All Patients With Drug-Eluting Stents? All Patients With Drug-Eluting Stents Need at Least 12 Months of Dual Antiplatelet Therapy	Circulation	Editorial
12	Byrne	2015	Bioresorbable Drug-Eluting Stents: An Immature Technology in Need of Mature Application	JACC: Cardiovascular Interventions	Editorial

For peer review only

13	Capodanno	2015	What about the risk of thrombosis with bioresorbable scaffolds?	Eurointervention	Review
14	Capodanno	2015	Triple antithrombotic therapy in atrial fibrillation patients with acute coronary syndromes or undergoing percutaneous coronary intervention or transcatheter aortic valve replacement	Eurointervention	Editorial
15	Capodanno	2015	Impact of bridging with perioperative low-molecular-weight heparin on cardiac and bleeding outcomes of stented patients undergoing non-cardiac surgery	Thrombosis and Haemostasis	Article
16	Cassese	2015	Prolonged dual antiplatelet therapy after drug-eluting stenting: meta-analysis of randomized trials	Clinical Research in Cardiology	Article
17	Chow	2015	Drug-coated balloons: a novel advance in the percutaneous treatment of coronary and peripheral artery disease	Interventional Cardiology	Review
18	Cohen	2015	Long-term outcomes in high-risk patients with non-ST-segment elevation myocardial infarction	Journal of thrombosis and thrombolysis	Review
19	Collet	2015	Dual antiplatelet treatment after stenting–Authors' reply	The Lancet	Commentary
20	Colombo	2014	Dual Antiplatelet Therapy after Drug-Eluting Stents — How Long to Treat?	NEJM	Editorial
21	Cortese	2015	Drug-Coated Balloon angioplasty: an intriguing alternative for the treatment of Coronary Chronic Total Occlusions	International journal of cardiology	Letter
22	Costa	2015	Perspectives on the 2014 ESC/EACTS Guidelines on Myocardial Revascularization	Journal of cardiovascular translational research	Review
23	Costa	2015	Impact of clinical presentation on ischaemic and bleeding outcomes in patients receiving 6-or 24-month duration of dual-antiplatelet therapy after stent implantation: a pre-specified analysis from the PRODIGY (Prolonging Dual-Antiplatelet Treatment After Grading Stent-Induced Intimal Hyperplasia) trial	European Heart Journal	Article
24	Crea	2015	Sex differences in mechanisms, presentation and management of ischaemic heart disease	Atherosclerosis	Review
25	Cutlip	2014	Antiplatelet therapy after coronary artery stenting	UpToDate, Waltham, MA	Review

For peer review only

26	Curzen	2015	Prolonged antiplatelet therapy after drug-eluting stents	The Lancet	Commentary
27	de la Torre Hernandez	2015	Dual Antiplatelet Therapy for 6 Months vs 12 Months After New-generation Drug-eluting Stent Implantation: Matched Analysis of ESTROFA-DAPT and ESTROFA-2	Revista Española de Cardiología (English Edition)	Article
28	De Rango	2015	Dual Antiplatelet Therapy after Carotid Stenting: Lessons from'Big Brother'	European journal of vascular and endovascular surgery: the official journal of the European Society for Vascular Surgery	Editorial
29	Dhall	2014	Truth Vs hype	NEJM	Commentary
30	Dohan	2015	Duration of Dual Antiplatelet Therapy after Drug-Eluting Stents	NEJM	Commentary
31	Eisen	2015	Antiplatelet therapy: Defining the optimal duration of DAPT after PCI with DES	Nat Rev Cardiol	Others
32	Elmariah	2015	Extended duration dual antiplatelet therapy and mortality: a systematic review and meta-analysis	The Lancet	Article
33	Fanari	2015	Cost Effectiveness of Antiplatelet and Antithrombotic Therapy in The Setting of Acute Coronary Syndrome: current perspective and literature review	American Journal of Cardiovascular Drugs	Review
34	Fareed	2015	Antithrombotic therapy in 2014: Making headway in anticoagulant and antiplatelet therapy	Nature Reviews Cardiology	Review
35	Fiedler	2015	Duration of Triple Therapy in Patients Requiring Oral Anticoagulation After Drug-Eluting Stent Implantation The ISAR-TRIPLE Trial	Journal of the American College of Cardiology	Article
36	Genereux	2015	Stent Thrombosis and Dual Antiplatelet Therapy Interruption With Everolimus-Eluting Stents Insights From the Xience V Coronary Stent System Trials	Circulation: Cardiovascular Interventions	Article
37	Gilard	2015	Double Antiplatelet Therapy Duration: Standardize or Personalize?	Journal of the American College of Cardiology	Editorial

For peer review only

38	Gilchrist	2015	Vignettes of DES Failure	Catheterization and Cardiovascular Interventions	Editorial
39	Giustino	2015	Duration of Dual Antiplatelet Therapy After Drug-Eluting Stent Implantation: A Systematic Review and Meta-Analysis of Randomized Controlled Trials	Journal of the American College of Cardiology	Article
40	Gupta	2014	Balancing ischemia vs. bleeding-- Jury still out.	NEJM	Commentary
41	Gupta	2014	Dual antiplatelets :Walking on a tight rope	NEJM	Commentary
42	Habib	2015	Endothelialization of drug eluting stents and its impact on dual anti-platelet therapy duration	Pharmacol Res	Review
43	Henderson		Primecuts–This Week In The Journals	Clinical Correlations	Others
44	Hernandez	2015	2014 Update on Interventional Cardiology	Revista Española de Cardiología	Review
45	Huang	2015	Is the Duration of Dual Antiplatelet Therapy after Implantation of Drug-Eluting Stents the Longer the Better	Medical Principles and Practice	Letter
46	Husted	2015	Antithrombotic therapy for long-term secondary prevention of acute coronary syndrome in high-risk patients	Therapeutics and clinical risk management	Review
47	Huynh	2015	Antiplatelet therapy: Risks and benefits of extended DAPT after stenting	Nat Rev Cardiol	Others
48	Iqbal	2015	The year in cardiology 2014: coronary intervention	European Heart Journal	Review
49	Kumana	2015	Absolute benefits and harms of dual antiplatelet therapy after drug eluting stenting	Hong Kong Medical Journal	Article
50	Keaney	2015	Balancing the Risks and Benefits of Dual Platelet Inhibition	NEJM	Editorial

For peer review only

51	Kereiakes	2015	Efficacy and Safety of a Novel Bioabsorbable Polymer-Coated, Everolimus-Eluting Coronary Stent The EVOLVE II Randomized Trial	Circulation-Cardiovascular Interventions	Article
52	Kereiakes	2015	Antiplatelet therapy duration following bare metal or drug-eluting coronary stents: The dual antiplatelet therapy randomized clinical trial	JAMA	Article
53	Kirtane	2015	Should all stent patients have prolonged dual antiplatelet therapy?	JACC: Cardiovascular Interventions	Editorial
54	Kohno	2015	Report of the American Heart Association (AHA) Scientific Sessions 2014, Chicago	Circulation Journal	Commentary
55	Koppara	2015	Optical coherence tomography surveillance following drug-eluting stent implantation	Minerva Cardioangiologica	Review
56	Lavi	2015	Biodegredable stent platforms–Are we heading in the right direction?	Canadian Journal of Cardiology	Editorial
57	Lee	2015	Bleeding risks are in the eye of the beholder	ACP Journal Club	Commentary
58	Lee	2014	Dual Antiplatelet Therapy for Coronary Artery Disease	Circulation Journal	Review
59	Lemesle	2015	Dual antiplatelet therapy and non-cardiovascular mortality	The Lancet	Commentary
60	Lhermusier	2015	Prasugrel hydrochloride for the treatment of acute coronary syndromes	Expert opinion on pharmacotherapy	Review
61	Liou	2015	Optimal duration of dual antiplatelet therapy following drug-eluting stents implantation: A meta-analysis of 7 randomised controlled trials	International journal of cardiology	Article
62	Lipkin	2014	1 out of a hundred patient will benefit from extended dual Rx	NEJM	Commentary
63	Liu	2015	P2Y12 receptor inhibitors for secondary prevention of ischemic stroke	Expert opinion on pharmacotherapy	Review
64	Liu	2015	Percutaneous coronary intervention strategies and prognosis for graft lesions	Experimental and	Article

For peer review only

			following coronary artery bypass grafting	Therapeutic Medicine	
65	Madhavan	2015	Post-PCI Antithrombotic Therapy in Patients Requiring Long-Term Anticoagulation	Current cardiology reports	Review
66	Marrs	2015	Duration of Dual Antiplatelet Therapy after Drug-Eluting Stents	NEJM	Commentary
67	Matteau	2015	Balancing Long-Term Risks of Ischemic and Bleeding Complications after Percutaneous Coronary Intervention with Drug-Eluting Stents	The American journal of cardiology	Article
68	Matthews	2015	Persistence with secondary prevention medications after acute myocardial infarction: Insights from the TRANSLATE-ACS study	American Heart Journal	Article
69	Mauri	2015	Duration of Dual Antiplatelet Therapy after Drug-Eluting Stents -Author's reply	NEJM	Commentary
70	McKavanagh	2015	A Review of the Key Clinical Trials of 2014	Cardiology and therapy	Review
71	McMillan	2014	Nice slant	New England Journal of Medicine	Commentary
72	Mega	2015	Pharmacology of antithrombotic drugs: an assessment of oral antiplatelet and anticoagulant treatments	The Lancet	Review
73	Mehran	2015	DAPT Duration After DES: What Is the “Mandatory” Duration?	Journal of the American College of Cardiology	Editorial
74	Meneses	2014	About DAPT trial	New England Journal of Medicine	Commentary
75	Moschonas	2015	Protease-activated receptor-1 antagonists in long-term antiplatelet therapy. Current state of evidence and future perspectives	International journal of cardiology	Review

For peer review only

76	Mukherjee	2015	After drug-eluting stent placement, 6 months of dual antiplatelet therapy was noninferior to 12 months	Annals of Internal Medicine	Commentary
77	Navarese	2015	Optimal duration of dual antiplatelet therapy after percutaneous coronary intervention with drug eluting stents: meta-analysis of randomised controlled trials	BMJ	Article
78	Palmerini	2015	Mortality in patients treated with extended duration dual antiplatelet therapy after drug-eluting stent implantation: a pairwise and Bayesian network meta-analysis of randomised trials	The Lancet	Article
79	Papadimitriou	2015	Triple Antithrombotic Therapy: Is it Time to Drop the Aspirin?	Hospital Chronicles	Review
80	Parmar	2014	Error in Study Procedures!	NEJM	Commentary
81	Price	2015	The Optimal Duration of Dual Antiplatelet Therapy After Drug-Eluting Stent Implantation: Chasing a Mirage	Journal of the American College of Cardiology	Editorial
82	Raffoul	2015	Dual antiplatelet therapy duration after the placement of a drug-eluting stent: what are the data?	Current treatment options in cardiovascular medicine	Review
83	Rao	2015	The Conundrum of Reducing Ischemic and Bleeding Events After PCI∗	Journal of the American College of Cardiology	Editorial
84	Reejhsinghani	2015	Prevention of stent thrombosis: challenges and solutions	Vasc Health Risk Manag	Review
85	Rinfret	2015	Percutaneous Coronary Intervention: Finally Mature Enough	Journal of the American College of Cardiology	Editorial
86	Robbins	2015	Periprocedural management of aspirin during colonoscopy: a survey of practice patterns in the United States	Gastrointestinal endoscopy	Article

For peer review only

87	Rohla	2015	Double or triple antithrombotic combination therapy in patients who need anticoagulation and antiplatelet therapy in parallel	European Heart Journal- Cardiovascular Pharmacotherapy	Review
88	Ruparelia	2015	Dual antiplatelet therapy following drug-eluting stent implantation: how long is long enough?	Expert review of cardiovascular therapy	Editorial
89	Sabouret	2015	Dual antiplatelet therapy: optimal timing, management, and duration	European Heart Journal- Cardiovascular Pharmacotherapy	Review
90	Samardzic	2015	Temporal changes of platelet reactivity after coronary stenting–a thing to think about	American Journal of Cardiology	Commentary
91	Schiele	2015	mpact of prolonged dual antiplatelet therapy after acute myocardial infarction on 5-year mortality in the FAST-MI 2005 registry	International journal of cardiology	Article
92	Schulz-Schupke	2015	ISAR-SAFE: a randomized, double-blind, placebo-controlled trial of 6 vs. 12 months of clopidogrel therapy after drug-eluting stenting	European Heart Journal	Article
93	Secemsky	2015	Comparison of Short-and Long-Term Cardiac Mortality in Early Versus Late Stent Thrombosis (from Pooled PROTECT Trials)	The American journal of cardiology	Article
94	Shimohama	2015	Intrastent Thrombus - What You See Is What You Get?	Circulation Journal	Editorial
95	Simon	2015	Omeprazole, pantoprazole, and CYP2C19 effects on clopidogrel pharmacokinetic-pharmacodynamic relationships in stable coronary artery disease patients	European Journal of Clinical Pharmacology	Article
96	Sipahi	2015	Duration of Dual Antiplatelet Therapy after Drug-Eluting Stents	NEJM	Commentary
97	Sommer	2015	Stent Thrombosis: Current Management and Outcomes	Current treatment options in cardiovascular medicine	Review

For peer review only

98	Spencer	2015	Dual antiplatelets for 30 mo after drug-eluting stents reduced stent thrombosis and CV and cerebrovascular events.	ACP Journal Club	Commentary
99	Spencer	2015	Longer Versus Shorter Duration Dual-Antiplatelet Therapy After Drug-Eluting Stent PlacementA Systematic Review and Meta-analysisDuration of Dual-Antiplatelet Therapy After Drug-Eluting Stents	Annals of Internal Medicine	Article
100	Takeuchi	2015	Optimum duration of dual antiplatelet treatment could be decided using 64-MDCT: A new hint to treating patients with stents	IJC Heart & Vasculature	Others
101	Thomas	2015	The future of P2Y12 receptor antagonists	Platelets	Review
102	Tomoda	2015	Duration of Dual Antiplatelet Therapy after Drug-Eluting Stents	NEJM	Commentary
103	Toyota	2015	Meta-analysis of Long-term Clinical Outcomes of Everolimus-eluting Stents	The American journal of cardiology	Article
104	Tremmel	2015	Late breaking trials of 2014 in coronary artery disease: Commentary covering ACC, EuroPCR, SCAI, TCT, ESC, and AHA	Catheterization and Cardiovascular Interventions	Commentary
105	Tsoumani	2015	Evaluating the bioequivalence of clopidogrel generic formulations	Current medical research and opinion	Editorial
106	Valgimigli	2015	Duration of dual antiplatelet therapy after drug-eluting stent implantation: will we ever reach a consensus?	European Heart Journal	Editorial
107	Van de Werf	2015	The year in cardiology 2014: acute coronary syndromes	European Heart Journal	Review
108	Vetrovec	2015	Another Challenge for the Presumed Safety Advantage of Bare Metal Stents	Catheterization and Cardiovascular Interventions	Editorial
109	Vranckx	2015	Peri-procedural use of rivaroxaban in elective percutaneous coronary intervention to treat stable coronary artery disease. The XPLORER trial	Thrombosis and Haemostasis	Article
110	Waksman	2015	Do you still have an appetite for a short DAPT trial?	Cardiovascular Revascularization Medicine	Editorial

For peer review only

111	Watanabe	2015	Antiplatelet therapy discontinuation and the risk of serious cardiovascular events after coronary stenting: observations from the CREDO-Kyoto Registry Cohort-2	PLoS ONE	Article
112	Wiviott	2015	Clinical evidence for oral antiplatelet therapy in acute coronary syndromes	The Lancet	Review
113	Yamaji	2015	Long-term Outcomes after Coronary Stent Implantation in Patients Presenting with versus without Acute Myocardial Infarction (An observation from Coronary Revascularization Demonstrating Outcome Study-Kyoto Registry Cohort-2)	The American journal of cardiology	Article
114	Yang	2015	Current antiplatelet agents: place in therapy and role of genetic testing	Journal of thrombosis and thrombolysis	Review
115	Yeh	2015	Benefits and risks of extended duration dual antiplatelet therapy after PCI in patients with and without acute myocardial infarction	Journal of the American College of Cardiology	Article
116	Yeh	2015	Dual Antiplatelet Platelet Therapy Duration Following Coronary Stenting	Journal of the American College of Cardiology	Editorial
117	Yeh	2015	Dual antiplatelet therapy duration and mortality–Authors' reply	The Lancet	Commentary
118	Yeh	2015	Close encounters with errors of the second kind: evaluating risks and benefits of long-term dual antiplatelet therapy	European Heart Journal	Editorial

For peer review only

Appendix 2: Content of the scholarly and public attention surrounding the DAPT study by source (n = 425)

Category	Overall n=425	Scientific communication 118 (27.7)	News 12 (2.8)	Blogs 3 (0.7)	Facebook posts 189 (44.4)	Tweets 75 (17.6)	YouTube 8 (1.9)	DAPT Website 20 (4.7)
Text favourable about the prolonged treatment	81 (19.1)	28 (23.7)	9 (75.0)	-	9 (4.8)	11 (14.7)	(100)	16 (80.0)
Text uncertain, with inappropriate mention of mortality	19 (4.5)	13 (11.0)	3 (25.0)	2 (66.7)	-	-	-	1 (5.0)
Electronic link	151 (35.5)	-	-	-	113 (59.8)	38 (50.6)	-	-
Referenced with no message	13 (3.1)	1 (0.8)	-	-	10 (5.3)	2 (2.7)	-	-
Text uncertain, with no mention of mortality	100 (23.5)	37 (31.4)	-	1 (33.3)	48 (25.4)	13 (17.3)	-	1 (5.0)
Text uncertain, with appropriate mention of mortality	34 (8.0)	29 (24.6)	-	-	1 (0.5)	3 (4.0)	-	1 (5.0)
Text not favourable about the prolonged treatment	15 (3.5)	3 (2.5)	-	-	8 (4.2)	3 (4.0)	-	1 (5.0)
Text not favourable about the prolonged treatment and critical of the authors' interpretation	12 (3.0)	7 (6.0)	-	-	-	5 (6.7)	-	-

For peer review only

STROBE 2007 (v4) Statement—Checklist of items that should be included in reports of *cross-sectional studies*

Section/Topic	Item #	Recommendation	Reported on page #
**Title and abstract**	1	(*a*) Indicate the study's design with a commonly used term in the title or the abstract	1
		(*b*) Provide in the abstract an informative and balanced summary of what was done and what was found	2
**Introduction**			**4**
Background/rationale	2	Explain the scientific background and rationale for the investigation being reported	4, 5
Objectives	3	State specific objectives, including any prespecified hypotheses	5
**Methods**			**6**
Study design	4	Present key elements of study design early in the paper	6
Setting	5	Describe the setting, locations, and relevant dates, including periods of recruitment, exposure, follow-up, and data collection	6
Participants	6	(*a*) Give the eligibility criteria, and the sources and methods of selection of participants ***(no participants were involved in this study. Unit of study was the items disseminating DAPT study).***	7
Variables	7	Clearly define all outcomes, exposures, predictors, potential confounders, and effect modifiers. Give diagnostic criteria, if applicable	7
Data sources/ measurement	8*	For each variable of interest, give sources of data and details of methods of assessment (measurement). Describe comparability of assessment methods if there is more than one group	7, 8
Bias	9	Describe any efforts to address potential sources of bias	*No*
Study size	10	Explain how the study size was arrived at	6
Quantitative variables	11	Explain how quantitative variables were handled in the analyses. If applicable, describe which groupings were chosen and why	NA
Statistical methods	12	(*a*) Describe all statistical methods, including those used to control for confounding	8
		(*b*) Describe any methods used to examine subgroups and interactions	
		(*c*) Explain how missing data were addressed	
		(*d*) If applicable, describe analytical methods taking account of sampling strategy	
		(*e*) Describe any sensitivity analyses	
**Results**			9

For peer review only

Participants	13*	(a) Report numbers of individuals at each stage of study—eg numbers potentially eligible, examined for eligibility, confirmed eligible, included in the study, completing follow-up, and analysed (in flow diagram)	9, 17
		(b) Give reasons for non-participation at each stage (in flow diagram)	17
		(c) Consider use of a flow diagram	9
Descriptive data	14*	(a) Give characteristics of study participants (eg demographic, clinical, social) and information on exposures and potential confounders (Unit of study was the items disseminating DAPT study)	9
		(b) Indicate number of participants with missing data for each variable of interest	
Outcome data	15*	Report numbers of outcome events or summary measures	9, 10
Main results	16	(*a*) Give unadjusted estimates and, if applicable, confounder-adjusted estimates and their precision (eg, 95% confidence interval). Make clear which confounders were adjusted for and why they were included	9, 10
		(*b*) Report category boundaries when continuous variables were categorized	
		(*c*) If relevant, consider translating estimates of relative risk into absolute risk for a meaningful time period	
Other analyses	17	Report other analyses done—eg analyses of subgroups and interactions, and sensitivity analyses	NA
**Discussion**			11
Key results	18	Summarise key results with reference to study objectives	11
Limitations	19	Discuss limitations of the study, taking into account sources of potential bias or imprecision. Discuss both direction and magnitude of any potential bias	11, 12
Interpretation	20	Give a cautious overall interpretation of results considering objectives, limitations, multiplicity of analyses, results from similar studies, and other relevant evidence	11, 12
Generalisability	21	Discuss the generalisability (external validity) of the study results	11
**Other information**			12-14
Funding	22	Give the source of funding and the role of the funders for the present study and, if applicable, for the original study on which the present article is based	13

*Give information separately for cases and controls in case-control studies and, if applicable, for exposed and unexposed groups in cohort and cross-sectional studies.

Note: An Explanation and Elaboration article discusses each checklist item and gives methodological background and published examples of transparent reporting. The STROBE checklist is best used in conjunction with this article (freely available on the Web sites of PLoS Medicine at http://www.plosmedicine.org/, Annals of Internal Medicine at http://www.annals.org/, and Epidemiology at http://www.epidem.com/). Information on the STROBE Initiative is available at www.strobe-statement.org.

For peer review only
